# Pharmacist-led interventions at hospital discharge: a scoping review of studies demonstrating reduced readmission rates

**DOI:** 10.1007/s11096-024-01821-y

**Published:** 2024-12-09

**Authors:** Carole Weber, Carla Meyer-Massetti, Nicole Schönenberger

**Affiliations:** 1 Clinical Pharmacology and Toxicology, Department of General Internal Medicine, Inselspital–Bern University Hospital, 3010 Bern, Switzerland; 2 Institute of Primary Healthcare BIHAM, University of Bern, 3012 Bern, Switzerland; 3 Graduate School for Health Sciences, University of Bern, 3012 Bern, Switzerland

**Keywords:** Clinical pharmacy, Drug-related readmission, Hospital discharge, Pharmacist, Scoping review, Transition of care

## Abstract

**Background:**

Substantial numbers of hospital readmissions occur due to medication-related problems. Pharmacists can implement different interventions at hospital discharge that aim to reduce those readmissions. It is unclear which pharmacist-led interventions at hospital discharge are the most promising in reducing readmissions.

**Aim:**

This scoping review aimed to summarise pharmacist-led interventions conducted at hospital discharge that demonstrated a reduction in readmissions.

**Method:**

We searched the MEDLINE, EMBASE and CINAHL databases up to February 2024. We included studies that focused on pharmacist-led interventions at hospital discharge and reported significant readmission reductions. Two reviewers independently screened titles, abstracts and full texts. Data extracted included study characteristics, populations and the type of implemented pharmacist-led interventions along with the reduction in readmission rates achieved.

**Results:**

We included 25 articles for data synthesis. Many of the studies included either implemented at least two interventions concurrently or were part of broader programmes involving other healthcare professionals. The most common pharmacist-led interventions associated with reduced readmission rates included medication reconciliation, counselling and post-discharge follow-up by telephone. Follow-up primarily aimed to improve patients’ treatment adherence through education about their medications. Furthermore, many studies reported on multi-component interventions that began at hospital admission or during inpatient stays, not only at discharge.

**Conclusion:**

Successfully reducing readmissions through pharmacist-led interventions at hospital discharge suggests the effectiveness of a holistic approach incorporating multiple interventions. While these findings offer insights for pharmacists, further research should focus on conducting high-quality studies using a multifaceted approach to identify the most appropriate timing and combination.

**Supplementary Information:**

The online version contains supplementary material available at 10.1007/s11096-024-01821-y.

## Impact statements

- Pharmacist-led interventions at hospital discharge can reduce the risk of medication-related readmissions and therefore improving patient outcomes.
- A multicomponent approach—combining medication reconciliation, patient education, and follow-up—has proven effective in preventing readmissions, addressing key challenges in the transition of care.
- The research gaps identified highlight the need for standardised protocols and cost-effectiveness studies of pharmacist-led interventions to reduce medication-related readmissions.

## Introduction

Reducing hospital readmissions has become a priority for many healthcare systems as they lead to increased patient morbidity and mortality and, thus, an extra financial burden [1]. Readmissions are defined as any unplanned hospitalisation that occurs within a specified time period after the initial discharge, with the 30-day threshold being the most common [2]. Readmissions can be attributed to various factors, but medication-related problems (MRPs), also referred to as drug-related problems (DRPs) in the literature, are significant among them. The Pharmaceutical Care Network Europe (PCNE) defines an MRP as “an event or circumstance involving drug therapy that actually or potentially interferes with desired health outcomes” [3]. One systematic review showed that around 21% of all readmissions were attributable to MRPs, and a median of 69% of these were preventable [4].

During inpatient stays, medications are often managed by healthcare professionals, and medication regimens are almost always changed during hospitalisation [5]. This can pose significant challenges for patients after discharge. One study in the United Kingdom showed that 37% of all patients experienced MRPs within eight weeks of hospital discharge, with 81% of these categorised as severe [6]. MRPs causing readmissions include but are not limited to inappropriate prescribing, non-adherence to treatment and transition of care (TOC) problems [7–10]. Risk factors for medication-related readmissions include polypharmacy, the prescription of specific medication groups like diuretics, insulin or anticoagulants and, once again, non-adherence to treatment [11, 12]. In this context, pharmacist-led interventions at hospital discharge have emerged as strategies to address these MRPs, by focusing on preventable factors such as prescribing problems and non-adherence, and by improving TOC [13–15]. These interventions include medication reconciliation, patient education, follow-up and enhancing TOC by communicating more effectively with subsequent institutions and healthcare professionals [16–19]. Patient education involves providing tailored information to patients about their medications, including purpose, proper use and potential side effects, to improve understanding and adherence whereas follow-up includes post-discharge counselling, often via telephone or in-person, to assess adherence, manage side effects and address any medication-related concerns [20, 21]. Enhancing TOC by communicating refers to facilitating effective information exchange between healthcare providers and institutions to ensure complete medication management information at the subsequent point of care [22].

Pharmacist-led interventions, including medication reviews, have been shown to be effective in reducing hospital readmissions and improving patient outcomes. For example, systematic reviews by Daliri et al. and Bülow et al. demonstrated that these interventions during hospitalisations can significantly reduce readmission rates and adverse drug events [23, 24]. Several randomised controlled trials (RCTs) have researched how pharmacist-led interventions at hospital discharge affect readmissions, but findings have been inconsistent. Gillespie et al. used medication reconciliation, patient education and follow-up visits, resulting in a significant decrease of 16% in all-cause readmissions and 80% in medication-related readmissions [25]. The OPTIMIST trial combined medication reviews, care coordination and follow-up calls and also significantly reduced readmissions (HR 0.62 for 30-day and 0.75 for 180-day-readmissions) [26]. In contrast, the study by Gurwitz et al. focused on high-risk patients and integrated medication reviews and counselling but found no significant impact on readmissions [27]. Similarly, Kempen et al. included medication reviews and follow-up but did not observe a reduction in readmissions, indicating that the effectiveness of these interventions may depend on the specific intervention components and patient populations [25–28]. To the best of our knowledge, two published literature reviews have focused their investigations on different pharmacist-led interventions implemented at hospital discharge to reduce readmissions [29, 30]. However, these two reviews only included studies conducted in the USA, and they included studies regardless of whether those interventions had positive effects on readmission rates or not [29, 30]. Therefore, a comprehensive understanding of precisely which pharmacist-led interventions at hospital discharge are the most promising for reducing readmissions was still lacking.

### Aim

This scoping review aimed to systematically explore the existing literature on pharmacist-led interventions at hospital discharge that had demonstrated a positive impact on hospital readmissions. By synthesising the available evidence, this review could then inform pharmacists providing interventions at hospital discharge aimed at reducing readmissions and identify gaps that can inform future research.

## Method

### Information sources and search strategy

The reporting of this scoping review adhered to the preferred reporting items for systematic reviews and meta-analyses extension for scoping reviews (PRISMA-ScR) checklist [31]. The review protocol was not published separately but is available upon request from the corresponding author. We chose the scoping review methodology in order to provide a broad overview of pharmacist-led interventions that have effectively reduced readmissions and to identify publication trends and knowledge gaps.

To address our research question of “Which pharmacist-led interventions implemented during hospital discharge processes have effectively reduced readmissions?” we systematically searched the EMBASE, MEDLINE and CINAHL bibliographic databases. Using the Ovid interface for searching EMBASE and MEDLINE and EBSCOhost for searching CINHAL, our search strategy included articles from the databases’ inception dates up to the final search conducted on 9 February 2024 for EMBASE and MEDLINE, and 12 February 2024 for CINAHL. We supplemented this with a backward citation search of the articles retained in the online search.

The search strategy involved combining subject headings, including ‘readmissions’, ‘pharmacists’ and ‘hospital discharge’, along with searching titles and abstracts for these subject headings and their synonyms in the free text. This combination was achieved using the Boolean operator “AND”. Records in Ovid EMBASE were filtered to exclude conference materials. The three reviewers developed this search strategy together and optimised it through discussions with an expert from the University of Bern’s medical library. The final search strategy is available in Supplementary File 1. Records were deduplicated using Zotero 6.0 software (2006, Center for History and New Media at George Mason University, Fairfax, USA).

### Eligibility criteria

To be included in the review, publications needed to describe interventions conducted or initiated by pharmacists or pharmacy personnel, including pharmacy technicians, either at or shortly after hospital discharge. This inclusion criterion is referred to as “pharmacist-led interventions at hospital discharge”. These interventions should have demonstrated a statistically significant reduction in readmissions among adult patients (> 18 years old). Readmissions were defined as any hospitalisation within a specified timeframe following the initial discharge. We chose this approach to ensure a feasible and comprehensive overview of the literature, in line with the aim of a scoping review. A reduction was considered significant if the p-value was below 0.05, or if the 95% confidence interval for odds or hazard ratios did not include 1. We considered peer-reviewed journal articles published in English, German, Italian, French and Spanish.

We excluded publications if the intervention described was not pharmacist-led or was initiated by pharmacists outside the hospital setting. This excluded interventions by community pharmacists. However, interventions provided by community pharmacists who were part of the hospital staff or acting under hospital guidance, for example in community pharmacies maintained on hospital ground with access to inpatient information, were included. Articles focusing on interventions not implemented at hospital discharge (e.g. during admission or hospitalisation) were also excluded. Studies exclusively examining patients with specific diagnoses or procedures (e.g. only patients with heart failure or after a specific surgery) or specific medications (e.g. antimicrobial stewardship programmes) were also ineligible and excluded, as were conference materials, editorials, comments and literature reviews (further referred to as “wrong study type”). All other study types were included.

### Study selection, data extraction and the synthesis of results

Two reviewers independently screened all the titles and abstracts for inclusion and then extracted the data. Any discrepancies in the screening and data extraction processes were resolved through consensus and discussion, with the involvement of a third reviewer if necessary. One reviewer synthesised the data, and the second subsequently verified it.

A data extraction table, developed by the three reviewers, guided the extraction of relevant variables (Supplementary File 2). Two reviewers independently charted the data and resolved any disagreements through discussion. Extracted data items included first author, year of publication, country of origin, study population, population size, setting, objectives, methods, outcome measures, interventions described, results and authors’ conclusions. Subsequently, we charted each encountered intervention from the included studies and summarised which interventions were conducted by each study. We classified the interventions as described in the original studies. If the description of the interventions lacked detail, we categorised them strictly based on the provided information, as we focused on pharmacist-led interventions and could not verify the involvement of pharmacy personnel in other processes.

## Results

### Selection of studies

**Fig. 1:**
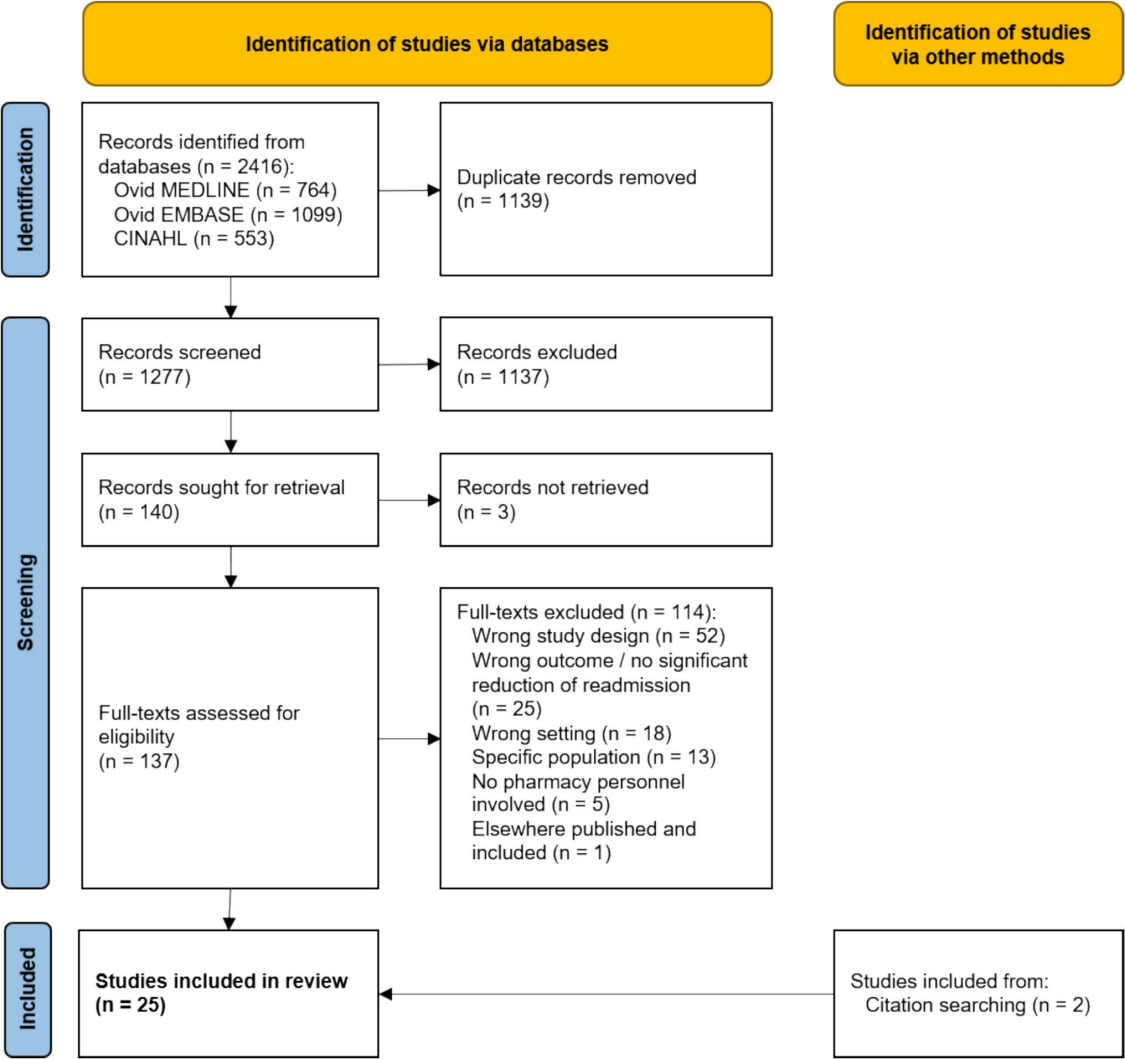
PRISMA flow diagram

After deduplication, 1277 records were screened and we requested 140 full-texts for review, of which three could not be retrieved due to unavailable records, leaving 137 full texts for eligibility assessment (Fig. 1). Using the pre-established inclusion and exclusion criteria, 23 studies were deemed eligible for inclusion. Two more studies were included via backward citation searching. The most common exclusion criteria during full-text screening were wrong study type (n = 52) (e.g. editorials or commentaries), wrong outcome (i.e. no readmission analysis or no reduction in readmissions, n = 25), wrong setting (n = 18) and wrong population (n = 13).

### Study characteristics

Study characteristics are summarised in Table 1, and a comprehensive data extraction table is presented in Supplementary File 2. Most of the studies were conducted in North America (n = 16), followed by Europe (n = 6), Asia (n = 2) and Australia (n = 1). We included a wide range of study designs as described by their authors, including RCTs (n = 8), quality improvement projects (n = 5), retrospective cohort studies (n = 3), pre–post studies (n = 3), non-randomised controlled trials (n = 2), prospective controlled studies (n = 2), a prospective cohort study (n = 1) and a clinical demonstration project (n = 1). The studies in Table 1 are listed chronologically from the most recent to the oldest publication. A minority of publications were published before 2010 (n = 6). The publication peak was in 2018 (n = 4), followed by 2020, 2016 and 2009 (n = 3 each). Notably, five of the six studies published before 2010 were RCTs or pilot RCTs. After 2010, only two RCTs met our inclusion criteria. Most studies focused on 30-day readmissions (n = 18) and specifically examined medication-related readmissions. The largest population involved a retrospective cohort study of 2253 patients. The smallest population consisted of 20 intervention patients included in a pilot RCT. In the RCTs included, interventions were administered to a maximum of 995 patients.

**Table 1:** Overview of the characteristics and readmission rate reductions described in the studies included in the review

First author (year, country)	Study design	Number of participants	Readmission rates
Lazaridis (2024, USA) [32]	Quality improvement project	1065 (all)	Lower readmission rates in two groups followed by population health pharmacists: 3% and 9% vs 23% (30 days), *P* < 0.01 9% and 14% vs 31% (60 days), *P*< 0.01 10% and 17% vs 35% (90 days), *P* < 0.01
Gallagher (2022, USA) [33]	Quality improvement project	1569 (intervention) 1813 (comparison)	7-day readmission rate fell from 7.6% to 5.8% 14- and 30-day readmission rates showed no difference
Fosnight (2020, USA) [34]	Quality improvement project	284 (intervention)	30-day readmission rate reductions of 20.4% (partial intervention) and 10.2% (full intervention) (*P* = 0.016) 30-day readmission rates fell from 21.0% (historical data) to 15.3% (intervention phase), 11.6% (with adherence interview) and 10.2% (all components)
Lam (2020, USA) [35]	Retrospective cohort study	2253 (intervention) 28,663 (comparison)	30-day readmission rate fell from 12.8% to 10.6% (*P* = 0.002) Non-significant in multivariate analysis
McConachie (2020, USA) [36]	Retrospective cohort study	177 (intervention) 197 (comparison)	30-day readmission rate fell from 21.7% to 11.4% in patients who received additional pharmacist interventions (65 out of 177 intervention patients), *P* = 0.04 15-day readmission rate showed no difference
Odeh (2019, UK) [21]	Prospective quasi-experimental study	211 (intervention) 211 (control; propensity-score matched)	30-day readmission rate fell by 10% (*P* < 0.001, OR 0.57) 90-day readmission rate fell by 15.2% (*P* = 0.021, OR 0.53) Patients who received three telephone calls showed a 20.6% reduction in 30-day readmission rate (*P* < 0.001, OR 0.22) and a 24.2% reduction in 90-day readmission rate (*P* = 0.012, OR 0.34) Patients who received just one or two calls showed no fall in readmission rate
Chiu (2018, CN) [37]	Prospective controlled study	108 (intervention) 104 (control)	30-day readmission rate fell from 29.1% to 13.2% (*P* = 0.005) 90-day readmission rate showed no difference
Ravn-Nielsen (2018, DK) [26]	Randomised controlled trial	498 (basic intervention) 497 (extended intervention) 503 (usual care)	Extended intervention reduced 30-day (HR 0.62, 95%CI = 0.46–0.84) and 180-day readmission rates (HR 0.75, 95%CI = 0.62–0.90) Basic intervention showed no reduction in readmission rates Changes in medication-related readmissions were not significant
Rottman-Sagebiel (2018, USA) [38]	Clinical demonstration project	388 (intervention) 1189 (comparison)	30-day readmission rate fell from 21.9% to 15.6% (*P* = 0.06)
Shanika (2018, LK) [39]	Non-randomised controlled trial	400 (intervention) 400 (control)	180-day medication-related readmission rate fell from 29.9% to 13.2% (*P* < 0.001)
Phatak (2016, USA) [40]	Randomised controlled trial	152 (intervention) 189 (control)	30-day readmission rate (including ED visits) fell from 39% to 24.8% (P = 0.01) Readmissions alone (without ED visits) showed no significant change Changes in medication-related readmissions were not significant
Rafferty (2016, USA) [41]	Prospective study (historical control)	384 (intervention) 1221 (control)	30-day readmission rate fell from 12.9% to 6.5% (*P* < 0.01) Re-presentations (including ED visits) fell at every time point (30 days, 60 days, 90 days (all *P* < 0.01) and 365 days (*P* = 0.02))
Zemaitis (2016, USA) [42 ]	Prospective study (historical control)	690 (admitted during the intervention period) 465 (received intervention)	30-day readmission rate fell from 26.2% to 15.8%, *P* = 0.009 (vs same period in previous year, among patients who actually received the intervention); from 24.7% to 18%, *P* = 0.009 (vs previous 6 months); and from 26.2% to 18%, *P* = 0.002 (same period in previous year in a per protocol analysis)
Balling (2015, USA) [43]	Quality improvement project	3143 (intervention period) 1058 (received intervention)	Readmissions per month: 25 (intervention period) vs 27.5 (control period), *P* = 0.0369 (discharges: 148 in intervention period vs 156.5 in control period, *P* = 0.0073)
Sanchez (2015, USA) [44]	Retrospective cohort study	277 (intervention) 124 (comparison)	30-day readmission rate (including ED visits) fell from 33.9% to 17.7%, *P* < 0.001 Readmissions alone (without ED visits) showed no significant change
Hutchison (2014, USA) [45]	Quasi-experimental pre–post study	384 (intervention) 452 (comparison)	90-day readmission rate fell from 51% to 39%, *P* < 0.001 30- and 60-day readmission rates showed no significant change
Pal (2013, USA) [46]	Prospective cohort study	537 (intervention) 192 (comparison)	30-day readmission rate fell from 26.0% to 16.8%, *P* = 0.006
Gardella (2012, USA) [47]	Quality improvement project	1624 (intervention period) 7335 (control period)	30-day readmission rate fell from 13.1% to 6.0%, *P* < 0.001 60-day readmission rate fell from 7.7% to 2.7%, *P* < 0.001 Adverse drug event-related readmissions and ED visits were 2.0% (intervention) vs 3.4% (control) (*P* < 0.0074) at 30 days and 0.6% (intervention) vs 2.5% (control) (*P* < 0.001) at 60 days
Sanchez Ulayar (2012, ES) [48]	Randomised controlled trial	50 (intervention) 50 (control)	30-day readmission rate fell from 24.4% to 7.3%, *P* < 0.05 60-day readmission rate fell from 31.7% to 7.3%, *P* < 0.01
Gillespie (2009, SE) [25]	Randomised controlled trial	199 (intervention) 201 (control)	365-day hospital readmission reduction (including ED visits): 16% (relative risk reduction, estimate 0.84, 95%CI = 0.720–0.99) 365-day readmission rate (without ED visits) showed no significant change 365-day medication-related readmission risk fell by 80% (relative risk reduction, estimate 0.20, 95%CI = 0.10–0.41
Jack (2009, USA) [49]	Randomised controlled trial	373 (intervention) 376 (control)	30-day hospital-use-rate ratio (including ED visits): 0.695, 95%CI = 0.515–0.937)
Koehler (2009, USA) [50]	Randomised controlled trial (pilot)	20 (intervention) 21 (control)	Composite of 30-day readmission and ED visit rates fell from 38.1% to 10.0% (*P* = 0.04) Composite of 60-day readmission and ED visit rate showed no significant change
Scullin (2007, UK) [51]	Randomised controlled trial	371 (intervention) 391 (control)	365-day readmission rate fell from 49.3% to 40.0%, *P* = 0.027
Crotty (2004, AU) [52]	Randomised controlled trial	56 (intervention) 54 (control)	60-day hospital use rate (including ED visits) among living patients fell from 22.7% to 11.4% (P = 0.035)
Al-Rashed (2002, UK) [53]	Non-randomised controlled trial	45 (intervention) 44 (control)	15–22-day readmission rate fell from 30% to 11%, *P* < 0.05 90-day readmission rate fell from 34% to 11%, *P* < 0.05

*AU* (Australia), *CN* (China), *DK* (Denmark), *ED* (emergency department), *ES* (Spain), *LK* (Sri Lanka), *SE* (Sweden), *UK* (United Kingdom of Great Britain and Northern Ireland), *USA* (United States of America)

### Pharmacist-led interventions at hospital discharge

A summary of the interventions implemented in the studies retained in our review is shown in Table 2. It also indicates whether the pharmacy personnel delivered their intervention as part of an interprofessional team including other healthcare professionals. Table 2 includes information on whether the intervention at discharge had been accompanied by prior interventions at hospital admission or during the inpatient stay. The most prevalent interventions at hospital discharge were patient counselling and education about their medications (n = 16), medication reconciliation (n = 10), medication plan development (n = 7), medication reviews (n = 7) and improving communication with the patient’s future primary care setting (n = 6). Twelve of the studies included provided post-discharge follow-up, the most common of which included patient counselling and treatment adherence measures (each n = 6), usually using follow-up telephone calls. All but one study [35] had provided either more than one intervention at hospital discharge or additional interventions at admission (n = 15) or during hospitalisation (n = 6). Details on the provided interventions at admission or during hospitalisations can be found in Supplementary File 2. Seven studies had integrated pharmacists into a larger interprofessional intervention. There were no discernible trends in the interventions over time, except that none of the studies conducted before 2010 provided medication reviews at hospital discharge. However, some of those studies had provided medication reviews during the inpatient stay. The most common combination of interventions was the medication reconciliation followed by patient education conducted in nine studies, with three of them also providing medication reviews.

**Table 2:** Summary of the interventions provided at hospital discharge. When a post-discharge follow-up was conducted, the specific interventions provided during this follow-up are shown in non-bold text in the subsequent lines that. Definitions of the interventions are provided in the table footnote

Interventions	Lazaridis (2024, USA) [32]	Gallagher (2022, USA) [33]	Fosnight (2020, USA) [34]	Lam (2020, USA) [35]	McConachie (2020, USA) [36]	Odeh (2019, UK) [21]	Chiu (2018, CN) [37]	Ravn-Nielsen (2018, DK) [26]	Rottman-Sagebiel (2018, USA) [38]	Shanika (2018, LK) [39]	Phatak (2016, USA) [40]	Rafferty (2016, USA) [41] [35]	Zemaitis (2016, USA) [42]
**Medication reconciliation**			X		X			X	X	X		X	X
**Medication review**	X	X					X		X	X		X	
**Medication plan development**								X			X		
**Patient education/counselling**	X		X		X		X		X	X	X	X	X
**Improving treatment adherence**							X		X				
**Communication with the patient’s primary care setting**								X				X	X
**Improving medication access**	X			X	X								
**Post-discharge follow-up**			X^a^		X	X		X	X		X		X
Medication reconciliation									X				
Medication review	X					X							
Patient education/counselling	X								X		X		
Improving treatment adherence	X					X		X	X		X		
Assistance organising follow-up									X				X
Communication with the patient’s primary care setting						X		X					
**Pharmacists were part of an interprofessional team**	X	X	X										
**Additional pharmacist intervention at admission**	X	X	X		X		X	X		X	X	X	X
**Additional pharmacist intervention during inpatient stay**	X		X							X			

Interventions	Balling (2015, USA) [43]	Sanchez (2015, USA) [44]	Hutchison (2014, USA) [45]	Pal (2013, USA) [46]	Gardella (2012, USA) [47]	Sanchez Ulayar (2012, ES) [48]	Gillespie (2009, SE) [25]	Jack (2009, USA) [49]	Koehler (2009, USA) [50]	Scullin (2007, UK)[51]	Crotty (2004, AU)[52]	Al-Rashed (2002, UK)[53]	Total
**Medication reconciliation**	X			X					X				10
**Medication review**			X										7
**Medication plan development**				X		X	X			X	X		7
**Patient education/counselling**	X			X		X	X		X	X		X	16
**Improving treatment adherence**	X											X	4
**Communication with the patient’s primary care setting**							X			X	X		6
**Improving medication access**	X									X			5
**Post-discharge follow-up**		X			X		X	X	X				12
Medication reconciliation		X											2
Medication review								X					3
Patient education/counselling					X			X	X				6
Improving treatment adherence							X						6
Assistance organising follow-up		X											3
Communication with the patient’s primary care setting		X						X					4
**Pharmacists were part of an interprofessional team**		X						X	X		X		7
**Additional pharmacist intervention at admission**	X				X		X		X	X			15
**Additional pharmacist intervention during inpatient stay**							X		X	X			6

*Medication reconciliation*: The process of creating an accurate list of all medications a patient is taking to ensure that medications are being added, changed or discontinued correctly across transitions of care; *Medication review*: Comprehensively evaluating medication regimens for appropriateness and safety; *Medication plan development*: Creating a personalised list for medication use, including dosing and scheduling and indicating changes made; *Patient education/counselling*: Providing information to patients about their medications, usage and potential side effects; *Improving treatment adherence*: Interventions aimed at increasing patient compliance with their prescribed treatment; *Communication with the patient’s primary care setting*: Sharing patient medication information with primary care providers to ensure continuity of care; *Improving medication access*: Facilitating patient access to necessary drugs; *Post-discharge follow-up*: Contacting patients after discharge to monitor, support medication use, improving treatment adherence and answer their questions; *Assistance organising follow-up*: Helping patients schedule post-discharge appointments with primary care providers

*AU* (Australia), *CN* (China), *DK* (Denmark), *ES* (Spain), *LK* (Sri Lanka), *SE* (Sweden), *UK* (United Kingdom of Great Britain and Northern Ireland), *USA* (United States of America)

^a^Conducted if needed

Among the eight RCTs analysed (see Table 3 for the results of this subgroup analysis), the most common interventions were completion of a medication plan (n = 6), patient counselling and education (n = 5), and improving communication with the patient’s future primary care providers (n = 6). Five of these RCTs included post-discharge follow-up, during which patient education and counselling, as well as adherence-improving interventions, were most frequently provided (each n = 3).

**Table 3:** Summary of the subgroup analysis of randomised controlled trials only. The interventions provided at hospital discharge are summarised. When a post-discharge follow-up was conducted, the specific interventions provided during this follow-up are shown in non-bold text in the subsequent lines. Definitions of the interventions are provided in the table footnote

Interventions	Ravn-Nielsen (2018, DK) [26]	Phatak (2016, USA) [40]	Sanchez Ulayar (2012, ES) [48]	Gillespie (2009, SE) [25]	Jack (2009, USA) [49]	Koehler (2009, USA) [49]	Scullin (2007, UK) [51]	Crotty (2004, AU) [52]	Total
**Medication reconciliation**	X					X			2
**Medication plan development**	X	X	X	X			X	X	6
**Patient education/counselling**		X	X	X		X	X		5
**Communication with the patient’s primary care setting**	X			X			X	X	4
**Improving medication access**							X		1
**Post-discharge follow-up**	X	X		X	X	X			5
Medication review					X				1
Patient education/counselling		X			X	X			3
Improving treatment adherence	X	X		X					3
Communication with the patient’s primary care setting	X				X				2
**Additional information**									
**Pharmacists were part of an interprofessional team**					X	X		X	3
**Additional pharmacist intervention at admission**	X	X		X		X	X		5
**Additional pharmacist intervention during inpatient stay**				X		X	X		3

*Medication reconciliation* The process of creating an accurate list of all medications a patient is taking to ensure that medications are being added, changed, or discontinued correctly across transitions of care; *Medication plan development* Creating a personalised list for medication use, including dosing and scheduling and indicating changes made; *Patient education/counselling* Providing information to patients about their medications, usage, and potential side effects; *Communication with the patient’s primary care setting* Sharing patient medication information with primary care providers to ensure continuity of care; *Improving medication access* Facilitating patient access to necessary drugs; *Post-discharge follow-up* Contacting patients after discharge to monitor, support medication use, improving treatment adherence and answer their questions; *Improving treatment adherence* Interventions aimed at increasing patient compliance with their prescribed treatment

*AU* (Australia), *DK* (Denmark), *ES* (Spain), *SE* (Sweden), *UK* (United Kingdom of Great Britain and Northern Ireland), *USA* (United States of America)

## Discussion

### Statement of key findings

This scoping review’s key findings highlight the diverse range of interventions used by pharmacists at hospital discharge that demonstrated reduced readmission rates. The most common interventions included medication reconciliation, patient counselling, medication reviews and post-discharge follow-up. Notably, most studies implemented multiple interventions or were part of broader interprofessional team, suggesting that a holistic approach is often necessary to reduce readmissions effectively. Most studies combined medication reconciliation with patient education.

### Strengths and weaknesses

To the best of our knowledge, this is the first scoping review to have comprehensively summarised pharmacist-led interventions at hospital discharge—from around the world—that were effective in reducing readmission rates. Nevertheless, some limitations should be considered when interpreting these results. One notable limitation is the heterogeneity in study designs, the interventions carried out and the different timeframes for readmissions, which precluded a quantitative synthesis of the results. The inclusion of study designs other than RCTs failed to control for potential confounding factors, and this may limit the generalisability of the results. Additionally, the inclusion criteria focused on studies demonstrating a reduction in readmissions, potentially introducing publication bias. Some of the studies retained failed to clearly describe whether and which pharmacist interventions the usual care group received, making it difficult to determine which pharmacist-led intervention was responsible for the reduced readmission rates. Furthermore, the studies retained often inadequately described their interventions, making it challenging to discern, for instance, which components of a medication review are the most effective in reducing readmissions. We acknowledge that our classification may not fully capture the sequence or complexity of activities performed due to limited descriptions in the original publications. To maintain methodological rigor and avoid bias, we did not infer additional steps, such as assuming that medication reconciliation preceded a medication plan development.

### Interpretation

These results highlight the significant role pharmacists can play in addressing MRPs and improving TOC at hospital discharge, both of which are issues that contribute significantly to readmissions. This aligns with previous research, including a meta-analysis by Rodrigues et al., which showed that pharmacy-supported TOC interventions positively influenced 30-day readmission rates (OR = 0.68; 95%CI = 0.68–0.75) [30]. Similarly, one systematic review found that 89.4% of the studies it included had demonstrated reduction in 30-day readmission rates due to pharmacist-led interventions during TOC [29]. Ensing et al. conducted a systematic review exclusively focused on RCTs, and studied the effect of pharmacist-led interventions during hospitalisation and post-discharge [54]. Their findings indicated significant variability in the effectiveness of these interventions, with strong evidence supporting the inclusion of medication reviews in comprehensive programmes [54]. Additionally, it highlighted the need for programmes that include medication reconciliation, patient counselling and the interprofessional collaboration across different TOC timepoints [54]. Our findings similarly highlight the importance of these interventions and the multicomponent approach, specifically at the point of hospital discharge. Research on discharge interventions in general has found that enhancing communication, providing patient education and adopting an interprofessional approach are effective in reducing readmissions, further aligning with the findings of this scoping review [55, 56].

Patient medication counselling at discharge was the intervention most frequently encountered by this review. Counselling improves medication adherence, a factor often associated with readmissions [57–59]. Indeed, a previous meta-analysis demonstrated that pharmacist-led medication reconciliation during TOCs reduced readmission rates by 19% [17]. Post-discharge follow-up, provided in 16 of the 25 studies retained, was also effective in reducing readmissions. This intervention’s effectiveness in reducing readmissions is highlighted in the meta-analysis by Rodrigues et al., where patient-centred follow-up was the only factor that reduced readmissions in a stratified analysis apart from the overall positive effect of all the interventions together [30]. Fragmented communication silos between healthcare settings and departments can impede TOC. Recent research by Marsall et al. showed that higher-quality TOCs correlated with fewer medication errors and improved patient health statuses [60]. This adds to the body of evidence that integrated, patient-centred follow-up can mitigate silo effects, enhance medication safety and thus reduce readmissions.

Most of the studies retained were published after 2010, suggesting an increasing recognition of the importance of pharmacist-led interventions in transitional care and potentially reflecting the growing emphasis on aiming for fewer hospital readmissions as a quality metric and a cost-saving measure.

The subgroup analysis of the eight RCTs included in this review yielded comparable results to those observed in the overall analysis. This further suggests that multicomponent interventions implemented at the time of hospital discharge may be an effective strategy for reducing readmissions, as all RCTs included in this review employed more than one intervention. The RCTs frequently included interventions such as patient education and counselling, improving communication, and post-discharge follow-up, which mirror the trends observed in the general analysis. It is noteworthy that none of the RCTs conducted medication reviews specifically at hospital discharge, although six included completion of a medication plan. It could be argued that a rigorously conducted medication plan development inherently involves elements of a medication review and reconciliation, as it typically requires an assessment of dosing, medication appropriateness and potential drug interactions. Furthermore, it is notable that medication reviews are frequently conducted during the hospitalisation period [23]. This may contribute to their underrepresentation at the point of discharge in the RCTs analysed because they have already been completed beforehand.

It is important to note that the present scoping review only included studies demonstrating successful reductions in readmission rates following pharmacist-led interventions at hospital discharge.

However, several RCTs have implemented similar interventions but have not found significant reductions in readmissions. For instance, the trial by Kempen et al. and the study by Gurwitz et al. used interventions like medication reconciliation, patient counselling and post-discharge follow-up, yet they failed to demonstrate any positive impact on readmission rates [27, 28]. Excluding these studies from our review does not diminish their importance or the quality of the research. Rather, it highlights the complexity of reducing readmissions and the potential influence of factors that go beyond pharmacist interventions alone, such as patients’ characteristics, healthcare system structures and study designs. Moreover, the combination, intensity and quality of the provided interventions may play a role in their effectiveness. Another important factor to consider is the statistical power of the studies. It is possible that some studies were underpowered to detect a statistically significant effect, even if the intervention was beneficial or on the other hand found significant results by chance. This lack of power could lead to false negatives and false positive results. Additionally, the method of patient selection may influence outcomes. For example, Gallagher et al. used a readmission risk score to prioritise patients who were most likely to benefit from their intervention, potentially leading to more effective results compared to studies that did not use such targeted approaches [33]. This suggests that interventions may need to be tailored not only to the type of intervention but also to specific patient populations to maximise their impact. Contrasting findings highlight the need for further research to elucidate the optimal combination, timing and strategy for implementing pharmacist-led interventions to maximise their effectiveness in reducing readmissions across diverse healthcare settings and patient populations.

### Further research

We have provided a comprehensive overview of studies providing pharmacist-led interventions at hospital discharge demonstrating reduced readmissions. Pharmacy policy-makers could use these findings to define or refine pharmacists’ interventions at hospital discharge aimed at reducing readmissions. Further research should aim to standardise and evaluate the effectiveness of specific intervention components or combinations, as well as explore the optimal timing and duration of interventions. Additionally, studies should investigate how different co-interventions can be combined or sequenced to enhance overall effectiveness. Furthermore, research should be conducted into which professions should collaborate with pharmacists and how in order to reduce readmissions. Studies investigating the cost-effectiveness and long-term sustainability of these interventions would be valuable for informing healthcare policies and resource allocation.

## Conclusion

This scoping review summarised studies, irrespective of their study design, that reported on pharmacist-led interventions implemented at hospital discharge demonstrating reduced readmission rates. Since all but one of the included studies provided either more than one intervention at hospital discharge or additional interventions at admission or during hospitalisations, a multi-component approach might be beneficial. The most commonly implemented interventions were medication reconciliation, patient education and post-discharge follow-up by telephone. By addressing MRPs and thus improving TOC, pharmacists can play a crucial role in reducing the financial burden of hospital readmissions and improving patients’ health outcomes.

## Supplementary Information

Below is the link to the electronic supplementary material. Below is the link to the electronic supplementary material.

Supplementary file1 (PDF 122 kb)

Supplementary file2 (PDF 617 kb)

## References

[1] Jencks SF, Williams MV, Coleman EA. Rehospitalizations among patients in the medicare fee-for-service program. N Engl J Med. 2009;360(14):1418–28. 19339721 10.1056/NEJMsa0803563

[2] Vest JR, Gamm LD, Oxford BA, et al. Determinants of preventable readmissions in the United States: a systematic review. Implement Sci. 2010;5:88. 21083908 10.1186/1748-5908-5-88 PMC2996340

[3] PCNE Classification for Drug-Related Problems V9.1. Pharmaceutical Care Network Europe Association; 2020. Available at: https://www.pcne.org/upload/files/417_PCNE_classification_V9-1_final.pdf. Accessed May 2020.

[4] El Morabet N, Uitvlugt EB, van den Bemt BJF, et al. Prevalence and preventability of drug-related hospital readmissions: a systematic review. J Am Geriatr Soc. 2018;66(3):602–8. 29468640 10.1111/jgs.15244

[5] Graabæk T, Terkildsen BG, Lauritsen KE, et al. Frequency of undocumented medication discrepancies in discharge letters after hospitalization of older patients: a clinical record review study. Ther Adv Drug Saf. 2019;10:2042098619858049. 31244989 10.1177/2042098619858049 PMC6580721

[6] Parekh N, Ali K, Stevenson JM, et al. Incidence and cost of medication harm in older adults following hospital discharge: a multicentre prospective study in the UK. Br J Clin Pharmacol. 2018;84(8):1789–97. 29790202 10.1111/bcp.13613 PMC6046489

[7] Cooper JB, Jeter E, Sessoms CJ. Rates and types of medication-related problems in patients rehospitalized within 30 days of discharge from a community hospital. J Pharm Technol. 2020;36(2):47–53. 34752555 10.1177/8755122519883642 PMC7047247

[8] Dalleur O, Beeler PE, Schnipper JL, et al. 30-day potentially avoidable readmissions due to adverse drug events. J Patient Saf. 2021;17(5):e379–86. 28306610 10.1097/PTS.0000000000000346

[9] Uitvlugt EB, Janssen MJA, Siegert CEH, et al. Medication-related hospital readmissions within 30 days of discharge: prevalence, preventability, type of medication errors and risk factors. Front Pharmacol. 2021;12: 567424. 33927612 10.3389/fphar.2021.567424 PMC8077030

[10] Whitaker AS, Cottrell WN. What proportion of unplanned re-presentations to an emergency department are medication related and preventable? J Pharm Pract Res. 2019;49(6):546–56.

[11] Linkens A, Milosevic V, van der Kuy PHM, et al. Medication-related hospital admissions and readmissions in older patients: an overview of literature. Int J Clin Pharm. 2020;42(5):1243–51. 32472324 10.1007/s11096-020-01040-1 PMC7522062

[12] Schönenberger N, Blanc AL, Hug BL, et al. Developing indicators for medication-related readmissions based on a Delphi consensus study. Res Social Adm Pharm. 2024;20(6):92–101. 38433064 10.1016/j.sapharm.2024.02.012

[13] Kripalani S, Roumie CL, Dalal AK, et al. Effect of a pharmacist intervention on clinically important medication errors after hospital discharge: a randomized trial. Ann Intern Med. 2012;157(1):1–10. 22751755 10.7326/0003-4819-157-1-201207030-00003 PMC3575734

[14] Walker PC, Bernstein SJ, Jones JNT, et al. Impact of a pharmacist-facilitated hospital discharge program: a quasi-experimental study. Arch Intern Med. 2009;169(21):2003–10. 19933963 10.1001/archinternmed.2009.398

[15] Miller D, Ramsey M, L’Hommedieu TR, et al. Pharmacist-led transitions-of-care program reduces 30-day readmission rates for Medicare patients in a large health system. Am J Health Syst Pharm. 2020;77(12):972–8. 32313954 10.1093/ajhp/zxaa071

[16] Kelly WN, Ho M-J, Bullers K, et al. Association of pharmacist counseling with adherence, 30-day readmission, and mortality: a systematic review and meta-analysis of randomized trials. J Am Pharm Assoc. 2021;61(3):340. 10.1016/j.japh.2021.01.028 33678564

[17] Mekonnen AB, McLachlan AJ, Brien JA. Effectiveness of pharmacist-led medication reconciliation programmes on clinical outcomes at hospital transitions: a systematic review and meta-analysis. BMJ Open. 2016;6(2): e010003. 26908524 10.1136/bmjopen-2015-010003 PMC4769405

[18] March KL, Peters MJ, Finch CK, et al. Pharmacist transition-of-care services improve patient satisfaction and decrease hospital readmissions. J Pharm Pract. 2022;35(1):86–93. 32945206 10.1177/0897190020958264

[19] Farley TM, Shelsky C, Powell S, et al. Effect of clinical pharmacist intervention on medication discrepancies following hospital discharge. Int J Clin Pharm. 2014;36(2):430–7. 24515550 10.1007/s11096-014-9917-x PMC4026363

[20] Al-Hashar A, Al-Zakwani I, Eriksson T, et al. Impact of medication reconciliation and review and counselling, on adverse drug events and healthcare resource use. Int J Clin Pharm. 2018;40(5):1154–64. 29754251 10.1007/s11096-018-0650-8

[21] Odeh M, Scullin C, Fleming G, et al. Ensuring continuity of patient care across the healthcare interface: telephone follow-up post-hospitalization. Br J Clin Pharmacol. 2019;85(3):616–25. 30675742 10.1111/bcp.13839 PMC6379220

[22] Witherington EMA, Pirzada OM, Avery AJ. Communication gaps and readmissions to hospital for patients aged 75 years and older: observational study. Qual Saf Health Care. 2008;17(1):71–5. 18245223 10.1136/qshc.2006.020842

[23] Bülow C, Clausen SS, Lundh A, et al. Medication review in hospitalised patients to reduce morbidity and mortality. Cochrane Database Syst Rev. 2023;1(1):Cd008986. 36688482 10.1002/14651858.CD008986.pub4 PMC9869657

[24] Daliri S, Boujarfi S, el Mokaddam A, et al. Medication-related interventions delivered both in hospital and following discharge: a systematic review and meta-analysis. BMJ Qual Saf. 2021;30(2):146–56. 32434936 10.1136/bmjqs-2020-010927

[25] Gillespie U, Alassaad A, Henrohn D, et al. A comprehensive pharmacist intervention to reduce morbidity in patients 80 years or older: a randomized controlled trial. Arch Intern Med. 2009;169(9):894–900. 19433702 10.1001/archinternmed.2009.71

[26] Ravn-Nielsen LV, Duckert M-L, Lund ML, et al. Effect of an in-hospital multifaceted clinical pharmacist intervention on the risk of readmission: a randomized clinical trial. JAMA Intern Med. 2018;178(3):375–82. 29379953 10.1001/jamainternmed.2017.8274 PMC5885912

[27] Gurwitz JH, Kapoor A, Garber L, et al. Effect of a multifaceted clinical pharmacist intervention on medication safety after hospitalization in persons prescribed high-risk medications: a randomized clinical trial. JAMA Intern Med. 2021;181(5):610–8. 33646267 10.1001/jamainternmed.2020.9285 PMC7922235

[28] Kempen TGH, Bertilsson M, Hadziosmanovic N, et al. Effects of hospital-based comprehensive medication reviews including postdischarge follow-up on older patients’ use of health care: a cluster randomized clinical trial. JAMA Netw Open. 2021;4(4):e216303. 33929523 10.1001/jamanetworkopen.2021.6303 PMC8087955

[29] Harris M, Moore V, Barnes M, et al. Effect of pharmacy-led interventions during care transitions on patient hospital readmission: a systematic review. J Am Pharm Assoc (2003). 2022;62(5):1477. 35718715 10.1016/j.japh.2022.05.017

[30] Rodrigues CR, Harrington AR, Murdock N, et al. Effect of pharmacy-supported transition-of-care interventions on 30-day readmissions: a systematic review and meta-analysis. Ann Pharmacother. 2017;51(10):866–89. 28599601 10.1177/1060028017712725

[31] Tricco AC, Lillie E, Zarin W, et al. PRISMA extension for scoping reviews (PRISMA-ScR): checklist and explanation. Ann Intern Med. 2018;169(7):467–73. 30178033 10.7326/M18-0850

[32] Lazaridis D, Partosh D, Ricabal LC, et al. Impact of a centralized population health pharmacy program on value-based Medicare patients 2003. J Am Pharm Assoc. 2024;64(1):146–53. 10.1016/j.japh.2023.09.008 37742742

[33] Gallagher D, Greenland M, Lindquist D, et al. Inpatient pharmacists using a readmission risk model in supporting discharge medication reconciliation to reduce unplanned hospital readmissions: a quality improvement intervention. BMJ Open Qual. 2022;11(1):e001560. 35241436 10.1136/bmjoq-2021-001560 PMC8896047

[34] Fosnight S, King P, Ewald J, et al. Effects of pharmacy interventions at transitions of care on patient outcomes. Am J Health Syst Pharm. 2020;77(12):943–9. 32374386 10.1093/ajhp/zxaa081

[35] Lam SW, Sokn E. Effect of pharmacy-driven bedside discharge medication delivery program on day 30 hospital readmission. J Pharm Pract. 2020;33(5):628–32. 30727808 10.1177/0897190019825961

[36] McConachie SM, Raub JN, Yost R, et al. Evaluation of a multidisciplinary approach to reduce internal medicine readmissions using a readmission prediction index. Am J Health Syst Pharm. 2020;77(12):950–7. 32382749 10.1093/ajhp/zxaa078

[37] Chiu P, Lee A, See T, et al. Outcomes of a pharmacist-led medication review programme for hospitalised elderly patients. Hong Kong Med J. 2018;24(2):98–106. 29302017 10.12809/hkmj176871

[38] Rottman-Sagebiel R, Cupples N, Wang CP, et al. A pharmacist-led transitional care program to reduce hospital readmissions in older adults. Fed Pract. 2018;35(12):42–50. 30766337 PMC6366589

[39] Shanika LGT, Jayamanne S, Wijekoon CN, et al. Ward-based clinical pharmacists and hospital readmission: a non-randomized controlled trial in Sri Lanka. Bull World Health Organ. 2018;96(3):155–64. 29531414 10.2471/BLT.17.198366 PMC5840627

[40] Phatak A, Prusi R, Ward B, et al. Impact of pharmacist involvement in the transitional care of high-risk patients through medication reconciliation, medication education, and postdischarge call-backs. J Hosp Med. 2016;11(1):39–44. 26434752 10.1002/jhm.2493

[41] Rafferty A, Denslow S, Michalets EL. Pharmacist-provided medication management in interdisciplinary transitions in a community hospital (PMIT). Ann Pharmacother. 2016;50(8):649–55. 27273678 10.1177/1060028016653139

[42] Zemaitis CT, Morris G, Cabie M, et al. Reducing readmission at an academic medical center: results of a pharmacy-facilitated discharge counseling and medication reconciliation program. Hosp Pharm. 2016;51(6):468–73. 27354748 10.1310/hpj5106-468 PMC4911987

[43] Balling L, Erstad BL, Weibel K. Impact of a transition-of-care pharmacist during hospital discharge 2003. J Am Pharm Assoc. 2015;55(4):443–8. 10.1331/JAPhA.2015.14087 26161488

[44] Sanchez GM, Douglass MA, Mancuso MA. Revisiting project re-engineered discharge (red): the impact of a pharmacist telephone intervention on hospital readmission rates. Pharmacotherapy. 2015;35(9):805–12. 26406772 10.1002/phar.1630

[45] Hutchison LJ, Mayzell GG, Bailey SC, et al. Impact of a discharge medication therapy management program in an extended care hospital. Consult Pharm. 2014;29(1):33–8. 24413012 10.4140/TCP.n.2014.33

[46] Pal A, Babbott S, Wilkinson ST. Can the targeted use of a discharge pharmacist significantly decrease 30-day readmissions? Hosp Pharm. 2013;48(5):380–8. 24421494 10.1310/hpj4805-380 PMC3839464

[47] Gardella JE, Cardwell TB, Nnadi M. Improving medication safety with accurate preadmission medication lists and postdischarge education. Jt Comm J Qual Patient Saf. 2012;38(10):452–8. 23130391 10.1016/s1553-7250(12)38060-4

[48] Sanchez Ulayar A, Gallardo Lopez S, Pons Llobet N, et al. Pharmaceutical intervention upon hospital discharge to strengthen understanding and adherence to pharmacological treatment. Farm Hosp. 2012;36(3):118–23. 21798784 10.1016/j.farma.2011.02.003

[49] Jack BW, Chetty VK, Anthony D, et al. A reengineered hospital discharge program to decrease rehospitalization: a randomized trial. Ann Intern Med. 2009;150(3):178–87. 19189907 10.7326/0003-4819-150-3-200902030-00007 PMC2738592

[50] Koehler BE, Richter KM, Youngblood L, et al. Reduction of 30-day postdischarge hospital readmission or emergency department (ED) visit rates in high-risk elderly medical patients through delivery of a targeted care bundle. J Hosp Med. 2009;4(4):211–8. 19388074 10.1002/jhm.427

[51] Scullin C, Scott MG, Hogg A, et al. An innovative approach to integrated medicines management. J Eval Clin Pract. 2007;13(5):781–8. 17824872 10.1111/j.1365-2753.2006.00753.x

[52] Crotty M, Rowett D, Spurling L, et al. Does the addition of a pharmacist transition coordinator improve evidence-based medication management and health outcomes in older adults moving from the hospital to a long-term care facility? results of a randomized, controlled trial. Am J Geriatr Pharmacother. 2004;2(4):257–64. 15903284 10.1016/j.amjopharm.2005.01.001

[53] Al-Rashed SA, Wright DJ, Roebuck N, et al. The value of inpatient pharmaceutical counselling to elderly patients prior to discharge. Br J Clin Pharmacol. 2002;54(6):657–64. 12492615 10.1046/j.1365-2125.2002.01707.x PMC1874498

[54] Ensing HT, Stuijt CCM, van den Bemt BJF, et al. Identifying the optimal role for pharmacists in care transitions: a systematic review. J Manag Care Spec Pharm. 2015;21(8):614–38. 26233535 10.18553/jmcp.2015.21.8.614 PMC10397897

[55] Becker C, Zumbrunn S, Beck K, et al. Interventions to improve communication at hospital discharge and rates of readmission: a systematic review and meta-analysis. JAMA Netw Open. 2021;4(8):e2119346. 34448868 10.1001/jamanetworkopen.2021.19346 PMC8397933

[56] Coffey A, Leahy-Warren P, Savage E, et al. Interventions to promote early discharge and avoid inappropriate hospital (re)admission: a systematic review. Int J Environ Res Public Health. 2019;16(14):2457. 31295933 10.3390/ijerph16142457 PMC6678887

[57] Bonetti AF, Bagatim BQ, Mendes AM, et al. Impact of discharge medication counseling in the cardiology unit of a tertiary hospital in Brazil: a randomized controlled trial. Clinics (Sao Paulo). 2018;73: e325. 29723341 10.6061/clinics/2018/e325 PMC5902758

[58] Marusic S, Gojo-Tomic N, Erdeljic V, et al. The effect of pharmacotherapeutic counseling on readmissions and emergency department visits. Int J Clin Pharm. 2013;35(1):37–44. 23007693 10.1007/s11096-012-9700-9

[59] Schönenberger N, Meyer-Massetti C. Risk factors for medication-related short-term readmissions in adults – a scoping review. BMC Health Serv Res. 2023;23(1):1037. 37770912 10.1186/s12913-023-10028-2 PMC10536731

[60] Marsall M, Hornung T, Bäuerle A, et al. Quality of care transition, patient safety incidents, and patients’ health status: a structural equation model on the complexity of the discharge process. BMC Health Serv Res. 2024;24(1):576. 38702719 10.1186/s12913-024-11047-3 PMC11069201

