# Supplementary material for: Pharmacist-led interventions at hospital discharge: a scoping review of studies demonstrating reduced readmission rates
Source: Int J Clin Pharm. 2024 Dec 9;47(1):15–30. doi: 10.1007/s11096-024-01821-y (PMC11741998; doi:10.1007/s11096-024-01821-y)
Supplement: Supplementary file 1 — Supplementary file1 (PDF 122 kb) [file 11096_2024_1821_MOESM1_ESM.pdf]

Supplementary File 1: Search Strategies

A scoping review of pharmacist-led interventions at hospital discharge that reduced readmissions

International Journal of Clinical Pharmacy

Corresponding author:
Nicole Schönenberger
Clinical Pharmacology and Toxicology
Department of General Internal Medicine
Inselspital–Bern University Hospital
Anna-von-Krauchthal-Weg 7
CH-3010 Bern
nicole.schoenenberger@insel.ch

#	Query	Results of 09.02.2024
1	exp Hospital discharge/ or Transitional Care/ or discharge*.ti,ab. or ((transition* or transfer*) adj2 care).ti,ab. or ((patient* or hospital) adj2 release*).ti,ab. or ((hospital or care*) adj2 interface).ti,ab.	646’714
2	Pharmacist/ or Clinical Pharmacist/ or Hospital Pharmacist/ or Pharmacy Technician/ or Hospital Pharmacy/ or Clinical Pharmacy/ or Medication therapy management/ or Evidence-based Pharmacy/ or (pharmacy* or pharmacist* or pharmacies).ti,ab. or (pharmaceutical adj2 (advice or care or service*)).ti,ab.	227’237
3	Hospital Readmission/ or (Readmission? or Re-admission? or Readmit$ or Re-admit$ or Rehospitali$ or Re-hospitali$).ti,ab. or ((repeat* or recurrent) adj2 (admission* or admitted or hospitali$)).ti,ab. or (hospital adj2 (revisit$ or return$)).ti,ab.	142’469
4	1 and 2 and 3	2519
5	4 not (conference abstract or conference paper or "conference review" or editorial or erratum or letter or note).pt.	1099

Conduct search in Ovid Embase

#	Query	Results of 09.02.2024
1	Patient discharge/ or Patient transfer/ or Hospital to home transition/ or discharge*.ti,ab. or ((transition* or transfer*) adj2 care).ti,ab. or ((patient* or hospital) adj2 release*).ti,ab. or ((hospital or care*) adj2 interface).ti,ab.	370’337
2	Pharmacists/ or Medication Therapy, Management/ or Pharmacy Service, Hospital/ or Evidence-based Pharmacy Practice/ or Pharmacy Technicians/ or (pharmacy* or pharmacist* or pharmacies).ti,ab. or (pharmaceutical adj2 (advice or care or service*)).ti,ab.	97’096
3	Patient Readmission/ or (Readmission? or Re-admission? or Readmit$ or Re-admit$ or Rehospitali$ or Re-hospitali$).ti,ab. or ((repeat* or recurrent) adj2 (admission* or admitted or hospitali$)).ti,ab. or (hospital adj2 (revisit$ or return$)).ti,ab.	67’820
4	1 and 2 and 3	764

Conduct search in Ovid Medline

#	Query	Results of 12.02.2024
1	((MH "Patient Discharge+") OR (MH "Continuity of Patient Care+") OR (TI (discharge*) OR AB (discharge*)) OR (TI ((transition* OR transfer*) N1 (care)) OR AB ((transition* OR transfer*) N1 (care))) OR (TI ((patient* OR hospital) N1 (release*)) OR AB ((patient* OR hospital) N1 (release*))))	131’697
2	((MH "Pharmacists") OR (MH "Pharmacy Technicians") OR (MH "Pharmacy Service") OR (MH "Medication Management") OR (TI pharmacist* OR AB pharmacist*) OR (TI pharmacies OR AB pharmacies) OR (TI pharmacy* OR AB pharmacy*) OR (TI ((pharmaceutical) N1 (advice OR care OR recommendation? OR service*)) OR AB ((pharmaceutical) N1 (advice OR care OR recommendation? OR service*))))	51’666
3	((MH "Readmission") OR (TI Readmission# OR AB Readmission#) OR (TI Re-admission# OR AB Re-admission#) OR (TI Readmit* OR AB Readmit*) OR (TI Re-admit* OR AB Re-admit*) OR (TI Rehospitali* OR AB Rehospitali*) OR (TI Re-hospitali* OR AB Re-hospitali*) OR (TI ((repeat* OR recurrent) N1 (admission*OR admitted OR hospitali*)) OR AB ((repeat* OR recurrent) N1 (admission*OR admitted OR hospitali*))) OR (TI (hospital N1 (revisit* OR return*)) OR AB (hospital N1 (revisit* OR return*))))	31’236
4	1 and 2 and 3	553

Conduct search in EBSCOhost CINAHL
