# Supplementary material for: Pharmacist-led interventions at hospital discharge: a scoping review of studies demonstrating reduced readmission rates
Source: Int J Clin Pharm. 2024 Dec 9;47(1):15–30. doi: 10.1007/s11096-024-01821-y (PMC11741998; doi:10.1007/s11096-024-01821-y)
Supplement: Supplementary file 2 — Supplementary file2 (PDF 617 kb) [file 11096_2024_1821_MOESM2_ESM.pdf]

Supplementary File 2: Data Extraction Table

A scoping review of pharmacist-led interventions at hospital discharge that reduced readmissions

International Journal of Clinical Pharmacy

Corresponding author:

Nicole Schönenberger

Clinical Pharmacology and Toxicology

Department of General Internal Medicine

Inselspital–Bern University Hospital

Anna-von-Krauchthal-Weg 7

CH-3010 Bern

nicole.schoenenberger@insel.ch

Reference (author, year, country)	Study design and population	Objectives	Methods and outcome measures	Interventions	Results	Discussion and conclusion
Lazaridis et al.<br>2024<br>USA<br>[1]	Retrospective analysis.<br><br>1'065 Medicare patients (11.2020 – 05.2021).	Providing comprehensive post discharge medication support and resolve medication therapy problems as well as readmissions during the 90-day risk period by using a tele-health pharmacy programme.	Uniform workflows, a pharmacy task force and a dual pharmacy team approach with population health registered nurses were established.<br><br>Statistical analyses:<br>Descriptive statistics, chi-squared test, Kruskal-Wallis test and multivariate regression models were used to assess differences after stratifying patients into 4 groups:<br>- Group 1: control, nurse services only.<br>- Group 2: nurse services and transition of care (TOC) pharmacist services.<br>- Group 3: nurse services and population health pharmacist services.<br>- Group 4: nurse services, TOC pharmacist and population health pharmacist services.<br><br>Outcomes:<br>- Medication therapy services.<br>- Readmission rates at 30-day, 60-day and 90-day intervals.	Discharge (TOC pharmacist):<br>- Discharge medication review and education.<br><br>Postdischarge/population health pharmacy services – phone call after 7 days:<br>- Comprehensive medication review.<br>- Medication therapy problem identification and resolution.<br>- Education/Re-education performed.<br>- Adherence and barriers assessed and supported (regarding costs, understanding and transportation).<br><br>Admission (TOC pharmacist):<br>- Admission medication clarification.<br>Inpatient stay (TOC pharmacist):<br>- Intake/barriers assessment.<br><br>Population health pharmacists collaborated with onsite TOC pharmacists, population health registered nurses, community pharmacies and health care professionals for medication therapy resolution.	- Groups with population health pharmacy services had significantly lower 30-, 60- and 90-day readmission rates compared to the control group (P < 0.01; 23% vs 3% and 9% (30 days), 31% vs 9% and 14% (60 days), 35% vs 10% and 17% (90 days).<br>- Groups without population health pharmacy services did not exhibit statistically significant reductions in 30-, 60- or 90-day readmissions (P = 0.73, 0.77, 0.8, respectively).	The programme improved patient outcomes, resolved medication therapy problems and reduced readmission rates.<br>The programme's success underscores the importance of interprofessional collaboration.<br>Expansion of TOC pharmacist interventions with population health pharmacist services was necessary to observe a statistically significant difference between control and intervention groups.
Gallagher et al.<br>2022<br>USA<br>[2]	Quality improvement interventional study.<br><br>1'569 high-risk adult general medicine patients (discharged between 01.10.2018 and 29.02.2020).	Using the Epic® readmission risk model to identify high-risk patients for the clinical pharmacists to intervene on by reducing medication errors at discharge and achieve reductions in unplanned readmissions.	High-risk patients were selected using Epic© readmission risk model (the highest 25% were selected) and 3 comparative cohorts were created:<br>- Group 1: Intervention group, high-risk patients with pharmacist involvement.<br>- Group 2: Historical controls, high-risk patients discharged before the intervention period, no pharmacist involved.<br>- Group 3: Concurrent controls, high-risk patients discharged during the intervention period when no clinical pharmacist was available.<br><br>Statistical analyses:<br>- Standardised mean differences, multivariable logistic regression and Kaplan-Meier curves.<br><br>Outcomes:<br>- Readmission rates of 7, 14 and 30 days.<br>- Targeted review of the number and kind of interventions the clinical pharmacists have performed.	Discharge (pharmacist):<br>- Pharmacists reviewed the discharge medication reconciliation that was conducted by physicians. The review process included:<br>• Accuracy and completeness by comparing home medications with inpatient orders and new orders at discharge.<br>• Medication dosing and frequency (including renal and hepatic adjustments).<br>• Duration of therapy and days of supply for antimicrobial and short course medication such as opiates.<br>• Therapeutic duplications.<br>• Drug-drug interactions.<br>• Cost or financial barriers of certain medications.<br>• After visit summary accuracy and clarity.<br>- Findings and suggestions were communicated to the provider. Changes were at the providers' discretion.<br><br>Admission (pharmacy technician):<br>- Medication history.	- 7-day readmissions were reduced: OR to group 2 1.41 (95% CI [1.01, 1.97]) and OR to group 3 1.49 (95% CI [1.07, 2.07]). Readmission rate 5.8% in intervention group and 7.6% in both control groups.<br>- No significant difference in readmission rates within 14 and 30 days after discharge.	Involving pharmacists in the discharge medication reconciliation process decreased 7-day readmission rate.<br>Recommendations made by the pharmacists were often accepted.<br>7-day hospital readmission rates are hypothesised to be more influenced by hospital discharge programme, while later readmissions are more likely to be influenced by clinic practices or home interventions.
Fosnight et al.<br>2020<br>USA<br>[3]	Quality improvement project.<br><br>284 patients (01.2016).	Initiating a new transitions process on a medical/surgical unit and assess its effect on 30-day readmission rates and length of stay.	Intervention groups were compared to prior-year baseline metrics from the same months.<br>Additionally, readmission and length of stay data were compared between patients in whom all 8 components of the pharmacy intervention were completed vs those receiving only partial intervention.<br><br>Statistical analyses: SAS® (version 9.4):<br>descriptive analyses, chi-squared test and a 2-tailed 2-sample t-test.<br><br>Outcome measures:<br>- 30-day readmission rates.<br>- Length of stay.	Discharge (pharmacist):<br>- Medication reconciliation and medication counselling.<br>- Follow-up phone call within 72 hours if necessary (if the patient has medication-related issues).<br><br>Admission and hospitalization (pharmacist):<br>- Verification of medication history with patient/caregiver and pharmacy and/or physician office (conducted by pharmacy technician).<br>- Medication evaluation, adherence interview, clarification of history and reconciliation issues, provision of recommendations and medication counselling throughout the hospital stay.<br>- Resolution of medication adherence barriers and verification of affordability of new medications.<br><br>Other interventions were provided by an interprofessional group.	- Patients that received all components of the intervention had a lower 30-day readmission rate (10.2% vs 20.4%, P = 0.016).<br>- Compared with a readmission rate of 21.0% during the same months of the prior year, readmissions decreased to 15.3% with the intervention (no statistical values provided).<br>- A further decrease to 11.6% was noted when the adherence interview was completed.	Pharmacists and pharmacy technicians are vital team members in TOC. Adding a comprehensive pharmacy intervention led to nearly 10 medication change recommendations per patient, along with reduced length of stay and readmission rates, especially with the full pharmacy intervention.

Reference (author, year, country)	Study design and population	Objectives	Methods and outcome measures	Interventions	Results	Discussion and conclusion
Lam et al.<br>2020<br>USA<br>[4]	Retrospective cohort study.<br><br>30'916 adult patients (admitted between 01.09.2013 and 31.10.2015).<br>2'253 patients (7%) received the intervention;<br>28'663 patients (93%) received usual care.	Evaluating the effects of pharmacy-driven bedside discharge medication delivery (BDMD) on 30-day readmission rates.	Patients who declined the service were allocated to the control group.<br><br>Statistical analyses: SPSS® (version 24.0): P values of < 0.05 were considered statistically significant. Multivariable logistic regression, chi-square test, Mann Whitney U and Student's t-test were conducted.<br><br>Primary outcome:<br>- 30-day readmission rates.<br>Secondary outcomes (evaluated at day 14 and day 30):<br>- Emergency department (ED) visits.<br>- Ambulatory care visits.<br>- Composite of ED visits and readmissions.	Discharge (decentralised on-site pharmacy technicians):<br>- Processing of prescription and submission of insurance claims to participating outpatient pharmacies for dispensing.<br>- When completed, the prescriptions were delivered to the patient's room and any payment transactions were finalised.	Univariate analysis:<br>- 30-day readmission 10.6% (intervention) vs 12.8% (control), (P = 0.002).<br>- Reduction in 14-day- (P = 0.03) and 30-day composite ED visits and readmissions (P = 0.002).<br>- Non-significant reduction in 14-day- and 30-day ED visits and ambulatory care visits (P = 0.72).<br><br>Multivariate analysis:<br>- Results were not significant.	BDMD by pharmacy technicians was associated with a decrease in 30-day readmission rates. This effect was lost after adjustment for baseline patient characteristics. This might be because patients that declined BDMD (=control group) were likely more capable of obtaining their medication than intervention patients.
McConachie et al.<br>2020<br>USA<br>[5]	Retrospective cohort study.<br><br>374 patients.	Description of the integration of a 30-day hospital readmission prediction index into the electronic medical record and its impact on pharmacist interventions during TOC.	The intervention targeted high-risk patients (identified with the Hospital All-Cause Thirty Day Readmission Index (HATRIX)).<br><br>Statistical analyses: SPSS® and R®.<br>Chi-square and Mann-Whitney U tests were performed, as well as multivariable logistic regression and log-rank test. Alpha values of < 0.05 were considered significant.<br><br>Primary outcome:<br>- 30-day readmission rates.<br>Secondary outcomes:<br>- Between-group difference in 15-day readmission rate.<br>- Number and type(s) of pharmacist intervention(s).<br>- Time required for pharmacist intervention(s).	Discharge (pharmacist):<br>- Medication reconciliation.<br>- Prescription discharge "medication to bed" programme.<br>- Education on medication and/or administration device technique.<br>- Facilitation of prior authorisation of medications if necessary.<br><br>Postdischarge (pharmacist):<br>- Telephone follow-up.<br><br>Admission (pharmacist):<br>- Admission medication reconciliation.<br><br>Other TOC services were provided by a multidisciplinary team.	- Patients who received pharmacist interventions had a lower 30-day readmission rate (11.4% vs 21.7%, P = 0.04).<br>- No difference in 15-day readmission rates.<br><br>Patients that received "usual" multidisciplinary TOC intervention (not performed by pharmacists) had no reduction in 30-day readmission rates.	A multidisciplinary TOC team approach did not reduce the 30-day readmission rate on an internal medicine service. However, patients who received additional direct pharmacist interventions guided by a readmission prediction index had a reduced 30-day readmission rate.
Odeh et al.<br>2019<br>Northern Ireland<br>[6]	Pragmatic, prospective, quasi-experimental study.<br><br>211 adult patients with polypharmacy (≥ 10 prescribed medications for chronic illnesses) (15.02.2016 – 30.06.2016).	Implementing a pharmacist-led, post discharge telephone follow-up intervention and evaluating its impact on rehospitalisation parameters in patients with polypharmacy in comparison with a matched control group.	Integrative medication management service by clinical pharmacists was standard care in the study hospital.<br><br>Statistical analyses: SPSS® (version 23).<br>Cost benefit analysis was done for economic evaluation. Intervention patients were matched by propensity score techniques with a control group.<br><br>Primary outcome:<br>- 30-day readmission rates.<br>Secondary outcomes:<br>- 90-day readmission rates.<br>- Time to readmission to hospital (if readmitted).<br>- Length of hospital stay (during first readmission).<br>- Economic impact.<br>- Patient-centred outcomes: 3 questionnaires (self-reported adherence score, adherence-related beliefs about medicines and patient satisfaction).	Postdischarge (pharmacist):<br>3 Telephone calls: After 10 days, 1 month and the start of the third month.<br>- Evaluation of issues patients had with their treatment/illness (e.g., discussion of adherence, concerns, ability to manage the medicines, provision of practical individual advice to help patients overcome barriers to adherence).<br>- Interventions were divided into subgroups: Medication adherence, health promotion, adverse event management, medication-related challenges such as packaging, container and printed direction, medication supply/obtaining prescriptions, over the counter products, patient self-evaluated health status.<br>- Medication review was also conducted. Issues were resolved with other health care providers.	- 30-day readmission rate: 10% reduction (P < 0.001, OR 0.57).<br>- 90-day readmission rate: 15.2% reduction (P = 0.021, OR 0.53).<br>- Patients with 3 calls: 20.6% reduction in 30-day readmission rate (P < 0.001, OR 0.22) and 24.2% reduction in 90-day readmission rate (P = 0.012, OR 0.34).<br>- Patients with only 1 or 2 calls: no reduction observed.	Despite already having an integrated medication management service in place, pharmacist telephone follow-ups reduced 30- and 90-day readmissions.<br>Time to readmissions, length of hospital stay upon readmission, healthcare costs and patient-centred outcomes were also positively impacted.

Reference (author, year, country)	Study design and population	Objectives	Methods and outcome measures	Interventions	Results	Discussion and conclusion
Chiu et al.<br>2018<br>China<br>[7]	Prospective controlled study.<br><br>212 geriatric patients (> 65 years) (admitted from 12.2013 – 09.2014).	Investigating the effectiveness of a comprehensive pharmacist intervention on medication use and hospital readmission among a group of geriatric inpatients in Hong Kong.	Eligible patients were assigned to an intervention or control group according to the week day of admission (control: Monday – Thursday, intervention: Friday – Sunday).<br><br>Statistical analyses: SPSS® (version 17.0). Descriptive analyses, t-test and chi square tests were performed.<br><br>Primary outcome:<br>- Appropriateness of prescription measured by the medication appropriateness index (MAI).<br>Secondary outcomes:<br>- Acceptance rate by physicians.<br>- Number of subjects with unintended discrepancies.<br>- Patient satisfaction with the programme (only for those living at home).<br>- Unplanned hospitalisations 1 and 3 months after discharge.	Discharge (pharmacist):<br>- Medication review to check for appropriateness using the MAI with recommendations to physicians.<br>- Counselling: Improve medication knowledge, ensure proper use of medications and compliance. Explanation of changes to medication regimen, indications, untoward effects and when to seek medical advice, medication storage and administration.<br>- Written information: E.g., patient information leaflets. If needed, diagrams and pictorial schedules were made.<br>- Organisation of assistance (family member, external care services) if adherence issues were detected.<br><br>Admission (pharmacist):<br>- Medication reconciliation: Identify discrepancies.<br>- Medication review to check for appropriateness using MAI with recommendations to physicians.<br>- Pharmacist counselling (without the part about the changes).	- 30-day readmission rate significantly lower in the intervention group (13.2% vs 29.1%, P = 0.005).<br>- Readmission rates at 3 months were not statistically different.<br>- ED visits at 1 month and 3 months were not statistically different.	This study supports the use of a hospital-based clinical pharmacist to enhance appropriate use of medication in geriatric patients. Hospital readmissions at 1 month were significantly reduced. This effect was lost after 3 months. The reason might have been inadequate sample size. Alternatively, this might imply that pharmacist intervention needs to be continued after patient discharge in order to have a sustained effect.
Ravn-Nielsen<br>2018<br>Denmark<br>[8]	Randomised clinical trial, multicentre study (Odense Pharmacist Trial Investigating Medication Interventions at Sector Transfer OPTIMIST).<br><br>1'499 adult patients with polypharmacy (≥ 5 medications).	Determine whether a multifaceted pharmacist intervention based on medication review, motivational interview and follow-up can reduce the number of short- and long-term readmissions and ED visits.	Patients admitted to the ED were randomised 1:1:1 into three groups: Usual care, basic intervention and extended intervention. After the medication review, patients in the intervention group underwent another randomisation to the basic or extended intervention group.<br><br>Statistical analyses: Stata software®<br>Unadjusted Cox proportional hazards regression, chi-squared test and multinomial logistic regression. 0.05 was defined as bilateral significance level.<br><br>Primary outcomes:<br>- Composite readmission or ED visits within 180 days.<br>- Readmissions within 30 or 180 days and ED visits within 180 days.<br>Secondary outcomes:<br>- All-cause mortality.<br>- Medication-related* mortality.<br>- Medication-related* readmissions within 30 and 180 days.<br>*: If an adverse drug reaction or dose-related therapeutic failure was present.	Discharge (hospital pharmacist):<br>- Medication reconciliation including a motivation interview with the patient and a summary of changed doses, new medications, discontinuations, administration, adverse drug events, adherence and costs.<br>- Medication-related problems and a summary outlining changed doses, new medications and medication therapy discontinuations was sent to the primary care provider (and nursing home if applicable).<br><br>Postdischarge (hospital pharmacist):<br>- If medication changes occurred: Follow-up call to primary care provider and caregiver (and outpatient pharmacy if deemed necessary) approximately 3 workdays after discharge.<br>- Telephone follow-up with patient at 1 week and 6 months after discharge.<br><br>Admission / Hospitalization (hospital pharmacist):<br>- Medication review.<br><br>Basic intervention: Medication review at admission and proposal of changes.<br>The medication reviews and patient interviews were followed up in collaboration with primary care providers.	- Reduced 30-day (HR 0.62, 95% CI [0.46, 0.84]) and 180-day readmission rate (HR 0.75, 95% CI [0.62, 0.90]).<br>- Composite of readmissions and ED visits (HR 0.77, 95% CI [0.64, 0.93]).<br>- Nonsignificant decrease in number of medication-related readmissions (basic intervention P = 0.89, extended intervention P = 0.11) within 30 days (95% CI [0.30, 1.09]) and within 180 days (95% CI [0.59, 1.08]).<br>- Readmissions could not be reduced significantly with the basic intervention (P = 0.5).	A multifaceted clinical pharmacist intervention may reduce the number of ED visits and hospital readmissions.
Rottman-Sagebiel et al.<br>2018<br>USA<br>[9]	Clinical demonstration project.<br><br>1'577 patients ≥ 65 years old (05.2013 – 09.2013 and 04.2014 – 03.2015): 1'189 control patients and 388 intervention patients.	Improving the quality of care for older veterans transitioning from hospital to home by implementing a new TOC programme. The goal was to reduce polypharmacy, inappropriate prescribing and 30-day readmissions.	12 or more medications were used as a cut-off for polypharmacy.<br>Patients who were eligible but did not receive the intervention due to staff capacity were used as comparison group.<br><br>Statistical analyses:<br>Generalised estimating equations and logistic regression analysis were performed.<br><br>Outcomes:<br>- 30-day readmission rate.<br>- Number and kind of recommendations made by clinical pharmacy specialist.	Discharge (clinical pharmacy specialist):<br>- Interaction with patient about:<br>• Medication renewal/refill process, knowledge and need for support regarding medication management.<br>• Medication review, medication reconciliation, patient education and assessment of medication adherence.<br>• Geriatric assessment on abilities and risk with the current medication (cognitive and falls screening).<br>- Recommendations regarding appropriateness of therapy (and barriers to medication adherence) using Beers and START/STOPP guidelines.<br><br>After discharge (clinical pharmacy specialist):<br>- Telephone follow up: Medication reconciliation, rectify medication errors, patient education, facilitating follow-	- Reduced 30-day readmission rate: 15.6% (TOC group) and 21.9% (control group); OR 0.74 (95% CI [0.54, 1.0], P = 0.06).<br>- After covariate adjustment: OR 0.54 (95% CI [0.32, 0.90], P = 0.02).	This clinical pharmacy specialist intervention reduced 30-day readmissions, stopped unnecessary medications and corrected medication errors and discrepancies in older patients discharged from hospital to home.

Reference (author, year, country)	Study design and population	Objectives	Methods and outcome measures	Interventions	Results	Discussion and conclusion
				up appointments, inquiring about concerns, receipt of newly prescribed medications and need for assistance.		
Shanika et al. 2018 Sri Lanka [10]	Non-randomised controlled trial.<br><br>715 patients with chronic non-communicable diseases (recruited from 03.13 – 09.13).	Assessing if a ward-based clinical pharmacy service resolving medication-related problems improved medication appropriateness at discharge and prevented medication-related hospital readmissions.	Statistical analyses: SPSS® (version 21)<br>Chi-squared and t-tests were performed. Level of significance = $P \leq 0.05$.<br><br>Outcomes:<br>- Identified medication-related problems.<br>- Appropriateness of prescribing at discharge (MAI).<br>- 6-month medication-related hospital readmissions.<br>- Direct costs of medication-related hospital readmissions.	Discharge (clinical pharmacist):<br>- Medication review and reconciliation: Discrepancies (deletion, additions, changes), identification of medication-related problems. Findings were recorded and discussed with the health care team.<br>- Medication education: Verbal and written instructions on the safe administration of their medicines and a medication list was provided.<br><br>Admission/hospitalisation (clinical pharmacist):<br>- Medication history and medication reconciliation.<br>- Daily medication chart review.	Significant reduction of:<br>- Medication-related readmissions (29.9% vs 13.2%, $P < 0.001$) (self-reported,334 intervention- and 311 control patients were assessed).<br>- Readmissions due to non-reconciliation of medication (18.3% vs 2.3%, $P < 0.001$).<br>No significant reduction of readmissions due to non-compliance.	A ward-based clinical pharmacy service improved appropriate prescribing, reduced medication-related problems and readmissions for patients with chronic diseases.
Phatak et al. 2016 USA [11]	Randomised clinical trial (prospective, single-period, longitudinal).<br><br>278 patients discharged to home with high-risk medications or with ≥ 3 prescription medications upon discharge.	Assessing the impact of intensive pharmacist involvement in TOC.	Usual care consisted of discharge medication reconciliation and counselling by a physician or nurse as well as evaluation of pharmacotherapy plans and rectifying medication errors or safety-related concerns.<br><br>Statistical analyses: SAS®<br>Fischer exact, chi-square, student t-tests and multivariate logistic regression were performed.<br><br>Outcomes:<br>- Medication errors and adverse drug events.<br>- Patient's knowledge related to their medications (measured by HCAHPS score).<br>- 30-day all-cause inpatient readmissions and ED visits.<br>A pharmacist called 30 days after discharge to assess study endpoints.	Discharge (pharmacist or pharmacy student):<br>- Provision of discharge instructions, medication counselling and personalised medication plan (discussion of discrepancies with the physician).<br><br>Postdischarge (pharmacist or pharmacy student):<br>Phone calls at day 3, 14 and 30 after discharge to:<br>- Clarify the discharge plan, resolve unanswered questions or medication-related issues, identify and overcome barriers to adherence and facilitate access to medication (by contacting pharmacies or physicians).<br>- Confirm the medication regimen, assess side effects, new symptoms and changes in the therapy.<br>- Assess endpoints.<br><br>Admission (pharmacist):<br>Face-to-face medication reconciliation.	- Significant reduction of 30-day readmissions/ED visits (39% control vs 24.8% intervention ($P = 0.01$)).<br>- Adjusted OR for intervention group 0.55 (95% CI [0.32, 0.94]).<br>- Adjusted OR for readmissions alone (without ED visits): Not significant.<br>- Medication-related readmissions not significant: 24% control vs 23% intervention ($P = 1.0$).	The pharmacist interventions had positive impact on adverse drug events, medication errors, readmissions and ED visits.<br>Medication-related readmissions were similar between the groups, which could suggest the difference in readmissions being not solely due to the intervention, but also due to chance.
Rafferty et al. 2016 USA [12]	Prospective study with historical control.<br><br>384 intervention patients (23.09.2013 – 14.05.2014) and 1'221 control patients (01.09.2012 – 30.05. 2013).	Evaluating the impact of an inpatient TOC pharmacist on re-presentations following discharge.	The intervention group was compared with a historical control group who had been admitted to the same patient care units 1 year prior.<br><br>Statistical analyses: Chi-squared test or Fischer's exact-test, t-test, Mann-Whitney U test and logistic regression.<br><br>Primary outcome:<br>- 30-day re-presentation (ED visits and readmissions).<br>Secondary outcomes:<br>- 60-, 90- and 365-day re-presentation rates.<br>- Health care resource utilisation avoidance, projected annual cost savings and return on investment.	Discharge (TOC pharmacist):<br>- Discharge medication reconciliation review and resolution of discrepancies.<br>- Discharge education: On discharge medications and the follow-up appointment schedule.<br>- Communication with primary care providers: Discharge summary was sent to the primary care provider. If a pharmacist was embedded in the office, a list with medication changes and access or adherence challenges was sent.<br><br>Admission (TOC pharmacist):<br>- Review of medication history, medication reconciliation and resolution of discrepancies.<br>- Early introduction of the pharmacy services to the patient/caregiver and assessment of adherence to medications.	Reduction in 30-day re-presentations (11% absolute, 50.2% relative) (OR 0.43, 95% CI [0.30, 0.61]). These results were similar after adjusting (OR 0.44; 95% CI [0.31, 0.62]).<br><br>Relative risk reduction:<br>- 51% in 30-day ED visits ($P < 0.01$).<br>- 50% in 30-day readmissions ($P < 0.01$).<br>- 33.6%, 24%, 10.8%, in 60-, 90- and 120-days re-presentations ($P < 0.01$, $P < 0.01$, $P = 0.02$).	The incorporation of an inpatient pharmacist to an interdisciplinary team to focus on medication management had a positive impact on re-presentations at all follow-up time points (30, 60, 90 and 365 days). It was also shown to be financially favourable for the institution.

Reference (author, year, country)	Study design and population	Objectives	Methods and outcome measures	Interventions	Results	Discussion and conclusion
Zemaitis et al.<br>2016<br>USA<br>[13]	Prospective, historical control study.<br><br>465 patients admitted from 10.2011 – 03.2012.	Evaluating the impact of pharmacy-facilitated medication reconciliation and patient education model with post discharge follow-up on 30-day readmissions.	6 month intervention period were compared to preceding 6 and 12 months control period. The duration of the control periods were 6 months each.<br><br>Primary outcome:<br>- 30-day readmission rate.<br>The goal was to detect a 20% difference in readmission rates.<br>Secondary outcomes:<br>- Total number of pharmacist- identified medication reconciliation interventions.<br>- Total pharmacy resource utilisation.<br>- Identification of patients at high risk for readmission.	Discharge (pharmacist):<br>- Medication and disease state counselling.<br>- Medication reconciliation.<br>- Attendance of daily interdisciplinary discharge rounds.<br><br>Postdischarge (pharmacy technician):<br>- Phone call 24-72 hours after discharge to remind patients about their upcoming follow-up appointment and assess study outcomes.<br>All information gathered was communicated with outpatient pharmacies, primary physician's offices and/or insurance companies.<br><br>Admission (pharmacy technician/pharmacist):<br>- Best possible medication history (pharmacy technicians), medication reconciliation (pharmacist).	Reduction of the 30-day readmission rate:<br>- By 27% compared to the previous 6 months (18% vs 24.7%, P = 0.009).<br>- By 31% when compared to the same time period in the previous year (18% vs 26.2%, P = 0.002).<br>- Greatest reduction when compared to patients in the same time period in the previous year in a per protocol analysis (15.8% vs 26.2%, P = 0.0009).	Pharmacy-facilitated medication reconciliation and patient education of general internal medicine patients decreased 30-day readmission rates.
Balling et al.<br>2015<br>USA<br>[14]	Quality improvement project.<br><br>1'058 patients.	Assessing the impact of TOC pharmacist during hospital discharge.	A pharmacist coordinated patient discharges in 2 inpatient units with historically high readmission rates. Control patients received usual care which included medication reconciliation by physician and discharge education by physician and nursing staff.<br><br>Statistical analyses: Stata®<br>Chi-square or Fisher's exact-test and Wilcoxon rank-sum test were performed. $P < 0.05$ was considered statistically significant.<br><br>Outcomes:<br>- Monthly readmission rates.<br>- Medication interventions made by the pharmacist at discharge.	Discharge (TOC pharmacist):<br>- Patient counselling on medications and disease to increase adherence, assessment of adherence.<br>- Coordinating receiving prescriptions in advance for prior authorisation or delivered medication to the nurse before discharge, checking with the outpatient pharmacy if there are any barriers for dispensing (availability/coverage etc.). Resolving of patient's financial barriers.<br>- Interdisciplinary rounds to discuss barriers to patient discharges and assist in planning the discharge.<br>- Medication reconciliation.<br><br>Admission:<br>- Review of patient information including insurance coverage, medication adherence and medication history.	- Reduced readmission rate per month in the intervention year vs the control year (median 25 vs 27.5, P = 0.0369).<br>- More discharges per month in the control year (156.5 vs 148, P = 0.0073).<br><br>The data for readmission and discharges are for all patients, including those with no pharmacist involvement.	The intervention educated patients, resolved medications errors and insurance issues that may lead to adherence problems and led to a decrease in median readmissions per month. It cannot be concluded that the pharmacist was the only reason for this decrease as it was no possible to provide direct care for all patients.
Sanchez et al.<br>2015<br>USA<br>[15]	Retrospective chart review.<br><br>401 patients (discharged from 07.2012 to 05.2013).	Project RED (=reengineered discharge) reduced rehospitalisation significantly. Objective of this study was determining the impact of a pharmacist telephone intervention on the 30-day unplanned readmission rate.	Patients were stratified into 2 groups: Contacted/intervention and unable to contact/no intervention/control. All patients were educated by a discharge nurse educator.<br><br>Statistical analyses: Chi-squared and t-tests.<br><br>Primary outcome:<br>- 30-day unplanned all-cause hospital utilisation (ED visits and readmissions).<br>Secondary outcomes:<br>- Time from discharge to patient contact by a pharmacist.<br>- Number of pharmacist interventions.<br>- Time spent on phone calls.	Postdischarge (4 days after discharge):<br>- Telephone call by pharmacist: Review of clinical status, medication reconciliation including an evaluation of discharge and home medications (patients were questioned about indication, how they are taking them and if any side effects occurred), reminder of follow-up appointments, discussion of what to do if a problem occurred. The pharmacist asked open-ended questions to assess the patient's knowledge about their medications.<br>Identified discrepancies or problems were solved by contacting the primary care provider, discharge physician, pharmacy or through further discharge education.	- Unplanned hospital use 30 days after discharge was 17.7% (intervention) vs 33.9% (control group) (P < 0.001). The difference remained significant when adjusting for baseline characteristics (17.7% vs 29.9%, P = 0.01).<br>- No significant reduction of readmissions (P = 0.07).<br>- Significant reduction of ED visits (0.27 vs 0.519, P < 0.001).	A pharmacist telephone intervention as part of a comprehensive discharge protocol (including nurses) can have a positive impact on patients during the TOC process by reducing the incidence of unplanned hospital utilisation.
Hutchison et al.<br>2014<br>USA<br>[16]	Quasi-experimental pre-post study design.<br><br>801 patients (admitted from 01.2009 – 12.2009 (control) and 02.2010 – 01.2011 (intervention)).	Studying the feasibility and effectiveness of a discharge medication therapy management programme.	Participants: Mostly long-term patients who are medically stable but require intense, regular medical attention.<br><br>Statistical analyses: GraphPad QuickCalcs®.<br>Independent Student's t-test and uncorrected chi-square analysis were performed.<br><br>Outcomes:<br>- Readmissions within 30, 60 and 90 days.<br>- Length of stay, number of doses of maintenance medications at last day of stay, type of pharmacist interventions and acceptance rate by physicians.	Discharge (clinical pharmacist):<br>- Medication review 5 days before discharge (long-term care hospital).<br>- Interventions communicated via notes in the electronic health record to treating physician. If accepted, they were implemented within the next day.	- Significant reduction of 90-day readmission rate from 51% to 39% (P < 0.001).<br>- Reduction of 30-day and 60-day readmission rates were not statistically significant.	The clinical pharmacist interventions had a high acceptance rate and resulted in a decrease in 90-day readmissions. The process is simple and required approximately 30 minutes daily of the pharmacists' time, suggesting a highly cost-effective model.

Reference (author, year, country)	Study design and population	Objectives	Methods and outcome measures	Interventions	Results	Discussion and conclusion
Pal et al.<br>2013<br>USA<br>[17]	Prospective, non-randomised cohort study.<br><br>729 adult participants (discharged between 01.2007 and 29.10.2010).	Studying the impact of a pharmacist-based predischarge medication reconciliation and counselling programme on 30-day readmission rates and study if polypharmacy and problem medications are suitable screening criteria.	Intent was evaluating all patients, but patients discharged with ≥ 10 medications or problem medications (project BOOST criteria: anticoagulants, insulin, aspirin plus clopidogrel, digoxin and narcotics) were prioritised.<br><br>Statistical analyses: OpenEpi® (version 2.3.1.)<br>Fisher's exact-test was performed. P ≤ 0.05 was considered significant.<br><br>Primary outcome:<br>- 30-day readmission rate.<br>Secondary outcomes:<br>- Presence of polypharmacy.<br>- Problem medications and their relationship with observed 30-day readmission rate.	Predischarge/discharge (pharmacist):<br>- One-on-one counselling on high-risk medications using the teach-back method.<br>- Medication reconciliation: Verification of prior-to-admission prescription and nonprescription medications and detection of any discrepancies. Discrepancies were noted in the medical record and appropriate action was taken. Consistency of medication reconciliation upon discharge was ensured. Necessary follow-ups were communicated to the discharge team.<br>- Medication calendar: Generated from the medical records, instructions, where appropriate, were included.	- Significant reduction of 30-day readmission rate: 16.8% (intervention) vs 26.0% (control group) (P = 0.006).<br>- Absolute risk reduction was 9.2, number needed to treat was 10.8.<br>- In the pharmacy review group, adults ≥ 65 years had a significantly lower 30-day readmission rate compared to younger patients (P = 0.014).	Pharmacist medication reconciliation and counselling reduced 30-day readmission rates statistically significant.<br><br>The screening tool (polypharmacy and problem medications) helped to prioritise the intervention.
Gardella et al.<br>2012<br>USA<br>[18]	Pre-post study design (study design was not mentioned in the article).<br><br>10'174 patients (admitted between 10.2007 and 12.2010 or 01.2009 and 12.2010).	The project had 2 goals: Improving the accuracy of preadmission medication lists and enhancing patient education through telephone calls to patients most at risk for adverse drug events or readmission.	Medically complex patients were defined as ≥ 65 years with 1 additional criterion (e.g., ≥ 5 routine medications).<br><br>Statistical analyses:<br>Chi-square test.<br><br>Outcome measures:<br>- 30- and 60-day readmissions.<br>- Adverse drug events-related 30- and 60-day readmissions.<br>- 30- and 60-day revisits to the ED.	Postdischarge (pharmacists, pharmacy technicians):<br>- Education via telephone (pharmacist):<br>• Comparison of medications actually taken by the patient with those intended for use.<br>• Indications and potential side effects.<br>- Second call after 30 days (pharmacy technician):<br>• Follow-up.<br>• Medication-related questions, discrepancies or other problems are referred to the pharmacist for resolution.<br><br>Admission (pharmacy technicians):<br>Best possible medication history within 24 hours.	Significant reduction in:<br>- 30-day readmission rate: 6.0% (intervention) vs 13.1% (control group) (P < 0.001), OR 2.34.<br>- 60-day readmission rate: 2.7% (intervention) vs 7.7% (control group) (P < 0.001), OR 3.02.<br>- Adverse drug events-related readmissions and ED visits: 2.0% vs 3.4% (P < 0.0074) for 30 days and 0.6% vs 2.5% (P < 0.001) for 60 days.	The pharmacist intervention was associated with a statistically significant reduction in 30- and 60-day readmissions, adverse drug events-associated 30- and 60-day readmissions and 30- and 60-day ED visits.
Sanchez Ulayar et al.<br>2012<br>Spain<br>[19]	Experimental, controlled, randomised study.<br><br>100 patients with polypharmacy (≥ 5 medications (12.2009 – 01.2010).	Determine the effectiveness of a pharmacist intervention at hospital discharge on understanding and correct administration of medications, as well as effect on 30- and 60-day readmission.	Statistical analyses:<br>Descriptive analysis, analysis of the homogeneity and/or comparability of the groups, Chi-square or Fisher's exact-test and Mann Whitney U or Student's t-test were performed. P ≤ 0.05 was considered significant.<br><br>Primary outcome:<br>- Number of incorrect administration patterns. Assessed by telephone call 7 days after discharge (for all patients).<br>Secondary outcome:<br>- 30- and 60-day readmission rates.	Discharge:<br>- Medication counselling using a personalised medication plan (schedule of all medications to be taken with doses and general recommendations on the correct use of medications), usefulness of every medication was explained as well as how it should be administered.<br>- The importance of adhering to the guidelines is emphasised. The patients could address any doubts.	- 30-day readmissions: 3 (7.3%) in the intervention and 10 (24.4%) in the control group (P < 0.05).<br>- 60-day readmissions: 3 (7.3%) in the intervention group and 13 (31.7%) in the control group (P < 0.01).	The pharmacist intervention increased the percentage of patients who understood and took their medication correctly according to the medical prescription. Additionally, it reduced hospital readmissions.
Gillespie et al.<br>2009<br>Sweden<br>[20]	Randomised controlled study.<br><br>400 patients ≥ 80 years (recruited between 01.10.2005 and 30.06.2006).	Investigating the effectiveness of interventions performed by ward-based pharmacists in reducing morbidity and hospital use among older patients.	Control group received non-pharmacist, standard care (similar elements but less extensive as in the intervention group).<br><br>Statistical analyses: R®<br>Logistic, linear and poisson regression, cox proportional hazards models.<br><br>Primary outcome:<br>- Frequency of hospital visits (ED and readmissions; total and medication-related) during a 12-month follow-up period.<br>Secondary outcome:<br>- Cost of hospital care.	Discharge (pharmacist):<br>- Counselling.<br>- Discharge summary: Medication changes, monitoring needs, therapeutic goals and medication-related problems.<br>- Discharge letter is sent to the general practitioner.<br><br>Postdischarge (pharmacist):<br>- Telephone follow-up after 2 months: Ensure adequate home medication management, record additional changes, encourage patients to ask questions.<br><br>Admission/hospitalisation:<br>- Medication history: Various information sources used.<br>- Semistructured interview: Adherence, understanding of drug therapy regimen, perceived problems and adverse effects, use of over-the counter medications etc.<br>- Comprehensive medication review.<br>- Counselling on new medications.	- 16% reduction of hospital visits within 12 months (quotient 1.88 vs 2.24; estimate 0.84; 95% CI [0.720, 0.99]).<br>- 47% reduction in visits to the ED (quotient 0.35 vs 0.66; estimate 0.53; 95% CI [0.37, 0.75]).<br>- 80% reduction in medication-related readmissions (quotient 0.06 vs 0.32; estimate 0.20; 95% CI [0.10, 0.41]).<br>- No significant reduction in readmissions alone.	If implemented on a population basis, the addition a pharmacist to health care teams could lead to major reductions in morbidity and health care costs.

Reference (author, year, country)	Study design and population	Objectives	Methods and outcome measures	Interventions	Results	Discussion and conclusion
Jack et al<br>2009<br>USA<br>[21]	Randomised controlled trial.<br><br>749 English-speaking hospitalised adults (mean age 49.9 years).	Assessing the effects of an intervention designed to minimise hospital utilisation after discharge.	<u>Statistical analyses</u>: S-Plus® (version 8.0) and Stata® (version 10).<br>Poisson test, log-rank test, poisson regression (subgroup analysis) and a 2-sided significance test were performed. P values < 0.05 was considered statistically significant.<br><br><u>Primary outcome</u>:<br>- Hospital utilisation: Total number of 30-day ED visits and readmissions per participant.<br><u>Secondary outcomes:</u><br>- Self-reported preparedness for discharge.<br>- Rate of primary care follow-up visits.<br>- Knowledge of discharge diagnosis.	<u>After discharge (clinical pharmacist involvement):</u><br>- Telephone follow-up with patients 2-4 days after discharge (min. 3 attempts) to reinforce the discharge plan and review medications.<br>- Communication of issues to the primary care provider or nurse.<br><br>The pharmacist involvement was part of a larger programme (RED = reengineered discharge) where nurses arranged follow-up appointments, confirmed medication reconciliation and conducted individualised patient education.	- Hospital utilisation within 30 days: Incidence rate ratio 0.695 (95% CI [0.515, 0.937]), P = 0.009).<br>- After exclusion of 1 usual care patient with more than 8 hospital utilisation, the difference remained statistically significant (P = 0.028).<br>- Patients reached for intervention: 228 (62%).	The interprofessional programme reduced hospital utilisation, improved patient self-perceived preparedness for discharge and increased primary care provider follow-ups.
Koehler et al.<br>2009<br>USA<br>[22]	Randomised controlled pilot study.<br><br>41 medical high-risk patients (elderly, with diagnoses predisposed to recidivism).	Assessing the impact of a supplemental care bundle targeting high-risk elderly inpatients on hospital readmissions and/or ED visits 30 and 60 days after discharge.	Usual care included medication review by pharmacists, medication reconciliation at admission and discharge medication education by nurses.<br><br><u>Statistical analyses:</u> Prism® (version 5) and SPSS® (version 15)<br>Student t-test, Fisher's exact-test and log-rank test were performed. P values < 0.05 considered statistically significant.<br><br><u>Outcomes</u>:<br>- Length of stay, illness severity and unplanned hospital readmission or ED visits at 30 and 60 days after discharge.<br>- All patient follow-ups were completed at 01.09.2007.	<u>Discharge (pharmacist):</u><br>- Medication reconciliation and medication counselling.<br><br><u>Postdischarge (pharmacist)</u>:<br>- Follow-up call (5-7 days after discharge): Reinforce education, review of medication use and assessment of side effects. An action plan could be recommended.<br>The intervention group received additional care from study care coordinator.<br><br><u>Admission/hospitalisation</u>:<br>- Medication reconciliation.<br>- Daily medication review and education.<br>- Additional medication recommendations if indicated.	- 30-day readmissions and ED visits were reduced from 8 (38.1%) to 2 (10.0%) (P = 0.04).<br>- Difference in 60-day readmissions and ED visits was not statistically significant (P = 0.26).	The interprofessional elderly care bundle reduced readmissions and ED visits within 30, but not 60 days. This suggest that the intervention's influence was strongest closer to the hospital stay. Due to the study's small sample size, small differences could not be detected.
Scullin et al.<br>2007<br>Northern Ireland<br>[23]	Randomised controlled trial.<br><br>762 patients (202 intervention patients).	Developing an integrated medicines management and assess its effects on clinical and economic outcomes.<br><br>Medicines management aims to have an optimal use of medicines from prescribing to administration by the patient.	The patients were assigned to an integrated medicines management service group or usual care group.<br><br><u>Statistical analyses:</u> SPSS®<br>Logarithmic independent samples t-test, Kaplan-Meier survival analysis log-rank test and chi-square test (or Fisher's exact-test) were performed. P-values < 0.05 were considered statistically significant.<br><br><u>Primary outcome</u>:<br>- Length of stay.<br><u>Secondary outcomes</u>:<br>- Time to a further hospital admission and number of readmissions over a 12-month follow-up period.<br>- Health care practitioner satisfaction with the new model of care (using a satisfaction questionnaire).	<u>Discharge (pair of clinical pharmacist and pharmacy technician):</u><br>- Generation and authorisation of discharge prescription, assess which medications need dispensing.<br>- Provision of a medicines record sheet (contains dosage instructions, laboratory findings etc.) and of other relevant information (e.g., steroid cards).<br>- Final patient counselling.<br>- Transfer of information to general practitioner.<br><br><u>Admission (pair of clinical pharmacist and pharmacy technician):</u><br>- Best possible medication history, information about allergies, side-effects, adherence, storing of medication at home and solving discrepancies.<br><u>Hospitalisation (pair of clinical pharmacist and pharmacy technician):</u><br>- Daily medication review, inpatient monitoring and counselling with a focus on new or stopped medication, high-risk medications, use of devices etc.	- Decreased rate of 12-month readmission: 40% vs 49.3% (P = 0.027) (59.2% of intervention patients were not readmitted compared to 50.7% in the control group).<br>- Number needed to treat: 11.7.<br>- Intervention patients had a significantly longer time (262 days) to be readmitted compared to the normal care patients (242 days) (P = 0.0356).	The integrated medicines management service provided by clinical pharmacists and pharmacy technicians was effective to reduce readmissions and length of hospital stay. It also resulted in cost-effectiveness. The service can be used as a template in other settings.

Reference (author, year, country)	Study design and population	Objectives	Methods and outcome measures	Interventions	Results	Discussion and conclusion
Crotty et al. 2004 Australia [24]	Randomised, single-blind, controlled trial.<br><br>110 older adult patients (transitioning between 10.2002 and 07.2003).	Assessing the impact of a pharmacist transition coordinator on medication management and health outcomes in older adults undergoing a first time transfer from a hospital to a long-term care facility.	<u>Statistical analyses</u>: SPSS® (version 11.5) and Stata® (version 7.0)<br>Independent-samples t-tests, Mann-Whitney U tests and analysis of covariance were performed. Alpha was set to 0.01.<br><br><u>Primary outcome</u>:<br>- Quality of prescribing (measured by the MAI).<br><u>Secondary outcomes</u>:<br>- ED visits.<br>- Hospital readmissions.<br>- Adverse drug events, falls, worsening mobility or behaviours, increased confusion, worsening pain.<br>Outcomes were assessed at discharge and 8 weeks after by independent pharmacists.	<u>Discharge (transition pharmacist):</u><br>- Medication transfer summary: Transfer of medication information to the long-term care facilities (including nursing staff, family physician, community pharmacist) on the day of discharge. Information included changes to medications that had been made and aspects of medication management that required monitoring.<br>- Coordination of a community-pharmacist led medication review.<br>- Case conference (within 14 to 28 days after discharge): Transition pharmacist, family physician, community pharmacist, registered nurse at the long-term care facility. The transition pharmacist provided information concerning medication use and appropriateness.	- Among alive patients during the 8-week follow-up: 11.4% hospital usage in the intervention vs 22.7% in the control group (P = 0.035, RR 0.38, 95% CI [0.15, 0.99]).<br>- When all patients were considered (including those who died or did not complete the study), hospital usage was similar.	Intervention involving a pharmacist transition coordinator improved aspects of inappropriate medication use in patients with a first-time transition to a long-term care facility.<br>Similar rates of hospital usage when including all patients can be explained by the small sample size, leaving the study underpowered to detect secondary outcomes.
Al-Rashed et al. 2002 UK [25]	Non-randomised controlled trial (study design not mentioned in the article).<br><br>83 elderly patients (> 65 years) prescribed more than 4 medications.	Evaluating if the use of medication and discharge summaries, together with in-patient pharmaceutical counselling and a simple medicine reminder card, help the delivery of seamless pharmaceutical care.	Usual care patients received nurse-led medication education, medication information discharge summary sheets and a medicine reminders.<br><br><u>Statistical analyses:</u> Two tailed t-test, chi-squared test and Mann-Whitney test.<br><br><u>Outcomes</u>:<br>- Length of visit.<br>- Number of items prescribed, correct knowledge about medication use, dosage interval, dose and adherence.<br>- Readmissions.<br>Home visits were held at 15-22 days and 3 month after discharge to assess the outcomes. The general practitioner and community pharmacist were given envelopes from the patient to obtain feedback on the discharge system.	<u>Discharge (pharmacist):</u><br>- Medication counselling 24 hours before discharge: Indications, other uses, side effects, doses and dosage times were stressed with help of the medicine reminder card.<br>- Importance of adherence explained together with the consequences of over- and underuse. The teach back method was used.<br>- Medication discharge summary.	Significant reduction of the readmission rate:<br>- At visit 1: 5 patients (11%) in the intervention group vs 13 patients (30%) in the control group (P < 0.05).<br>- At visit 2: 3 patients (11%) vs 15 patients (34%) in the control group (P < 0.05).	Pharmacist-conducted medication counselling, together with a medication discharge summary and a medicine reminder card lead to better medication knowledge and adherence, reduced unplanned visits to the doctor and readmissions. The home visits improved health care outcomes.

Abbreviations: BDMD, bedside discharge medication delivery; CI, confidence interval; ED, emergency department; HR, hazard ratio; MAI, medication appropriateness index; OR, odds ratio; RR, risk ratio; SPSS, statistical product and service solutions; TOC, transition of care

References

1. Lazaridis D, Partosh D, Ricabal LC et al. Impact of a centralized population health pharmacy program on value-based Medicare patients. J Am Pharm Assoc (2003). 2024;64(1):146-53.
2. Gallagher D, Greenland M, Lindquist D et al. Inpatient pharmacists using a readmission risk model in supporting discharge medication reconciliation to reduce unplanned hospital readmissions: a quality improvement intervention. BMJ Open Qual. 2022;11(1).
3. Fosnight S, King P, Ewald J et al. Effects of pharmacy interventions at transitions of care on patient outcomes. Am J Health Syst Pharm. 2020;77(12):943-9.
4. Lam SW, Sokn E. Effect of Pharmacy-Driven Bedside Discharge Medication Delivery Program on Day 30 Hospital Readmission. J Pharm Pract. 2020;33(5):628-32.
5. McConachie SM, Raub JN, Yost R et al. Evaluation of a multidisciplinary approach to reduce internal medicine readmissions using a readmission prediction index. Am J Health Syst Pharm. 2020;77(12):950-7.
6. Odeh M, Scullin C, Fleming G et al. Ensuring continuity of patient care across the healthcare interface: Telephone follow-up post-hospitalization. Br J Clin Pharmacol. 2019;85(3):616-25.
7. Chiu P, Lee A, See T et al. Outcomes of a pharmacist-led medication review programme for hospitalised elderly patients. Hong Kong Med J. 2018;24(2):98-106.
8. Ravn-Nielsen LV, Duckert M-L, Lund ML et al. Effect of an In-Hospital Multifaceted Clinical Pharmacist Intervention on the Risk of Readmission: A Randomized Clinical Trial. JAMA Intern Med. 2018;178(3):375-82.
9. Rottman-Sagebiel R, Cupples N, Wang CP et al. A Pharmacist-Led Transitional Care Program to Reduce Hospital Readmissions in Older Adults. Fed Pract. 2018;35(12):42-50.
10. Shanika LGT, Jayamanne S, Wijekoon CN et al. Ward-based clinical pharmacists and hospital readmission: a non-randomized controlled trial in Sri Lanka. Bull World Health Organ. 2018;96(3):155-64.
11. Phatak A, Prusi R, Ward B et al. Impact of pharmacist involvement in the transitional care of high-risk patients through medication reconciliation, medication education, and postdischarge call-backs. J Hosp Med. 2016;11(1):39-44.
12. Rafferty A, Denslow S, Michalets EL. Pharmacist-Provided Medication Management in Interdisciplinary Transitions in a Community Hospital (PMIT). Ann Pharmacother. 2016;50(8):649-55.
13. Zemaitis CT, Morris G, Cabie M et al. Reducing Readmission at an Academic Medical Center: Results of a Pharmacy-Facilitated Discharge Counseling and Medication Reconciliation Program. Hosp Pharm. 2016;51(6):468-73.
14. Balling L, Erstad BL, Weibel K. Impact of a transition-of-care pharmacist during hospital discharge. J Am Pharm Assoc (2003). 2015;55(4):443-8.
15. Sanchez GM, Douglass MA, Mancuso MA. Revisiting Project Re-Engineered Discharge (RED): The Impact of a Pharmacist Telephone Intervention on Hospital Readmission Rates. Pharmacotherapy. 2015;35(9):805-12.
16. Hutchison LJ, Mayzell GG, Bailey SC et al. Impact of a discharge medication therapy management program in an extended care hospital. Consult Pharm. 2014;29(1):33-8.
17. Pal A, Babbott S, Wilkinson ST. Can the targeted use of a discharge pharmacist significantly decrease 30-day readmissions? Hosp Pharm. 2013;48(5):380-8.
18. Gardella JE, Cardwell TB, Nnadi M. Improving medication safety with accurate preadmission medication lists and postdischarge education. Jt Comm J Qual Patient Saf. 2012;38(10):452-8.
19. Sanchez Ulayar A, Gallardo Lopez S, Pons Llobet N et al. Pharmaceutical intervention upon hospital discharge to strengthen understanding and adherence to pharmacological treatment. Farm Hosp. 2012;36(3):118-23.
20. Gillespie U, Alassaad A, Henrohn D et al. A Comprehensive Pharmacist Intervention to Reduce Morbidity in Patients 80 Years or Older: A Randomized Controlled Trial. Arch Intern Med. 2009;169(9):894-900.
21. Jack BW, Chetty VK, Anthony D et al. A reengineered hospital discharge program to decrease rehospitalization: a randomized trial. Ann Intern Med. 2009;150(3):178-87.
22. Koehler BE, Richter KM, Youngblood L et al. Reduction of 30-day postdischarge hospital readmission or emergency department (ED) visit rates in high-risk elderly medical patients through delivery of a targeted care bundle. J Hosp Med. 2009;4(4):211-8.

23. Scullin C, Scott MG, Hogg A et al. An innovative approach to integrated medicines management. J Eval Clin Pract. 2007;13(5):781-8.
24. Crotty M, Rowett D, Spurling L et al. Does the addition of a pharmacist transition coordinator improve evidence-based medication management and health outcomes in older adults moving from the hospital to a long-term care facility? Results of a randomized, controlled trial. Am J Geriatr Pharmacother. 2004;2(4):257-64.
25. Al-Rashed SA, Wright DJ, Roebuck N et al. The value of inpatient pharmaceutical counselling to elderly patients prior to discharge. Br J Clin Pharmacol. 2002;54(6):657-64.
